# A Synthetic Lethality Screen Using a Focused siRNA Library to Identify Sensitizers to Dasatinib Therapy for the Treatment of Epithelial Ovarian Cancer

**DOI:** 10.1371/journal.pone.0144126

**Published:** 2015-12-04

**Authors:** Harsh B. Pathak, Yan Zhou, Geetika Sethi, Jeff Hirst, Russell J. Schilder, Erica A. Golemis, Andrew K. Godwin

**Affiliations:** 1 Department of Pathology and Laboratory Medicine, University of Kansas Medical Center, Kansas City, Kansas, United States of America; 2 Biostatistics and Bioinformatics Facility, Fox Chase Cancer Center, Philadelphia, Pennsylvania, United States of America; 3 Department of Biochemistry and Molecular Biology, Drexel University College of Medicine, Philadelphia, Pennsylvania, United States of America; 4 Department of Gynecologic Medical Oncology, Thomas Jefferson University, Philadelphia, Pennsylvania, United States of America; 5 Molecular Therapeutics Program, Fox Chase Cancer Center, Philadelphia, Pennsylvania, United States of America; 6 University of Kansas Cancer Center, Kansas City, Kansas, United States of America; Cedars-Sinai Medical Center, UNITED STATES

## Abstract

Molecular targeted therapies have been the focus of recent clinical trials for the treatment of patients with recurrent epithelial ovarian cancer (EOC). The majority have not fared well as monotherapies for improving survival of these patients. Poor bioavailability, lack of predictive biomarkers, and the presence of multiple survival pathways can all diminish the success of a targeted agent. Dasatinib is a tyrosine kinase inhibitor of the Src-family kinases (SFK) and in preclinical studies shown to have substantial activity in EOC. However, when evaluated in a phase 2 clinical trial for patients with recurrent or persistent EOC, it was found to have minimal activity. We hypothesized that synthetic lethality screens performed using a cogently designed siRNA library would identify second-site molecular targets that could synergize with SFK inhibition and improve dasatinib efficacy. Using a systematic approach, we performed primary siRNA screening using a library focused on 638 genes corresponding to a network centered on EGFR, HER2, and the SFK-scaffolding proteins BCAR1, NEDD9, and EFS to screen EOC cells in combination with dasatinib. We followed up with validation studies including deconvolution screening, quantitative PCR to confirm effective gene silencing, correlation of gene expression with dasatinib sensitivity, and assessment of the clinical relevance of hits using TCGA ovarian cancer data. A refined list of five candidates (*CSNK2A1*, *DAG1*, *GRB2*, *PRKCE*, and *VAV1*) was identified as showing the greatest potential for improving sensitivity to dasatinib in EOC. Of these, *CSNK2A1*, which codes for the catalytic alpha subunit of protein kinase CK2, was selected for additional evaluation. Synergistic activity of the clinically relevant inhibitor of CK2, CX-4945, with dasatinib in reducing cell proliferation and increasing apoptosis was observed across multiple EOC cell lines. This overall approach to improving drug efficacy can be applied to other targeted agents that have similarly shown poor clinical activity.

## Introduction

Ovarian cancer is the second most common gynecological cancer afflicting women in the United States [1]. With roughly 14,000 deaths and 22,000 new cases estimated annually, ovarian cancer has the highest mortality-to-new case ratio among all gynecologic malignancies [1]. There are three main types of ovarian tumors classified based on their tissue of origin (surface epithelium, stromal endocrine cells, and germ cells), with epithelial carcinomas accounting for >90% of ovarian malignancies [2]. These epithelial tumors are generally further divided based on their cellular morphology, the four most common subtypes being serous, endometrioid, clear cell, and mucinous, with each subtype having different pathogenesis, chemo sensitivities, and prognoses [2, 3]. Currently, the standard of care for women diagnosed with advanced stage disease is optimal debulking surgery followed by taxane- and platinum-based chemotherapy (generally paclitaxel or docetaxel in combination with carboplatin) [2]. Despite favorable response rates to this initial treatment, ~75% of the patients eventually experience a recurrence of a tumor that ultimately becomes treatment-resistant. Response to therapy is often less frequent in patients with recurrent disease and of shorter duration compared to initial therapy. Although improved in the last decade, the overall 5-year survival rate of patients with ovarian cancer is still only 44% [1]. However, as additional molecular characterizations of disease subtypes and a better understanding of their clinical biology and predictive biomarkers become available, personalized medicine can be provided to improve patient outcomes through the inclusion of targeted therapies [3–6].

Molecular targeted therapies are currently the major focus of clinical trials for the treatment of patients with recurrent ovarian cancer [7–11]. The Src-family kinases (SFKs), the most prominent member of the nine-member family being SRC kinase, are membrane associated non-receptor tyrosine kinases frequently overexpressed and activated in a variety of human cancers and cell lines [12], including a majority of late-stage serous, mucinous, and endometrioid epithelial ovarian tumors and EOC cell lines [13–16]. They play key roles in regulating signal transduction from cell surface receptors [17–19] and therefore, modulate many cellular functions including tumor progression and metastasis in a variety of human tumors and have received a renewed interest as potential targets for therapy [20–23]. Dasatinib is an orally administered ATP-competitive kinase inhibitor of the SFKs and the BCR-ABL fusion protein as well as other tumor-relevant kinases [24, 25]. Dasatinib is one of three treatments available to patients newly diagnosed with Philadelphia chromosome-positive chronic myeloid leukemia [26]. It continues to be investigated for anti-tumorigenic activity against a range of hematologic and solid tumors (http://www.clinicaltrials.gov/). We recently reported the results of a Gynecologic Oncology Group (GOG) sponsored Phase 2 clinical trial, GOG170M, evaluating dasatinib for the treatment of patients with recurrent or persistent EOC or primary peritoneal carcinoma [27]. Dasatinib showed minimal activity as a single agent in these patients [27], similar to the results from many previous GOG-170 series of Phase 2 clinical trials evaluating other targeted agents as monotherapies ([4], www.GOG.org).

In the vast majority of these single-agent trials, patient selection based on a particular biomarker was not done which may have improved the observed response rates. In addition, the dysregulation and integration of multiple survival pathways in ovarian tumors [4] make single-agent therapies prone to succumb to theses activated networks. Therefore, in the current study, our goal was to experimentally identify and validate second-site molecular target(s) that can help sensitize ovarian cancer cells to dasatinib and thereby improve its efficacy. To achieve this goal, we initially performed small interfering RNA (siRNA)-mediated gene silencing using a library targeting protein networks relevant to ovarian cancer and SFK function. This library was centered on receptor tyrosine kinases (RTKs) including epidermal growth factor receptor (EGFR) and HER2; their effectors SHC1 and SHC3; and three CAS-family proteins, NEDD9, BCAR1, and EFS, which directly bind and influence SFK and BCR-ABL, and in some cases have been shown to influence dasatinib response [28–31]. We used this library to identify synthetically lethal combinations that improve the sensitivity of EOC cells to dasatinib. We then systematically narrowed our hit list from the primary screen and identified several candidate dasatinib sensitizers (*CSNK2A1*, *DAG1*, *GRB2*, *PRKCE*, and *VAV1)*. Of these, *CSNK2A1* generated one of them more prominent sensitization effects in the siRNA screens and its expression levels were found to be predictive of dasatinib sensitivity in a panel of EOC cell lines. In addition *CSNK2A1* was overexpressed by almost 2-fold in patients with serous cystadenocarcinomas. Therefore, *CSNK2A1* was deemed to be the most promising target among the five hits for inhibition to enhance dasatinib activity. *In vitro* drug combination studies performed using dasatinib and CX4945 (silmitasertib), the first and only clinically relevant CK2 inhibitor [32], showed significant synergy across a panel of EOC cell lines in reducing proliferation and increasing apoptosis. The focused, systematic approach that we have taken in this study to identify second-site sensitizers to improve dasatinib efficacy can also be applied to other targeted agents that have similarly shown poor clinical activity.

## Results

### Identification of dasatinib-sensitizing hits

The primary screening of an EOC cell line was performed using a custom designed, siRNA library focused on targeting the signaling protein network centered on EGFR, HER2, SHC1, SHC3, NEDD9, BCAR1, and EFS. This custom library consisted of 1,276 siRNA duplexes targeting 638 human genes (a pool of two siRNAs per gene per well). The design and development of this network-based focused siRNA screening library was previously described by Astsaturov *et al*. [33]. We chose this library because it targeted many of the signaling proteins which act upstream, downstream, or in parallel with SFK, given its interplay with RTKs [34, 35] and its prominent role in facilitating signal transduction from RTKs [17–19], providing us with the most relevant and likely candidates to target in combination with inhibition of SFK.

**Fig 1:**
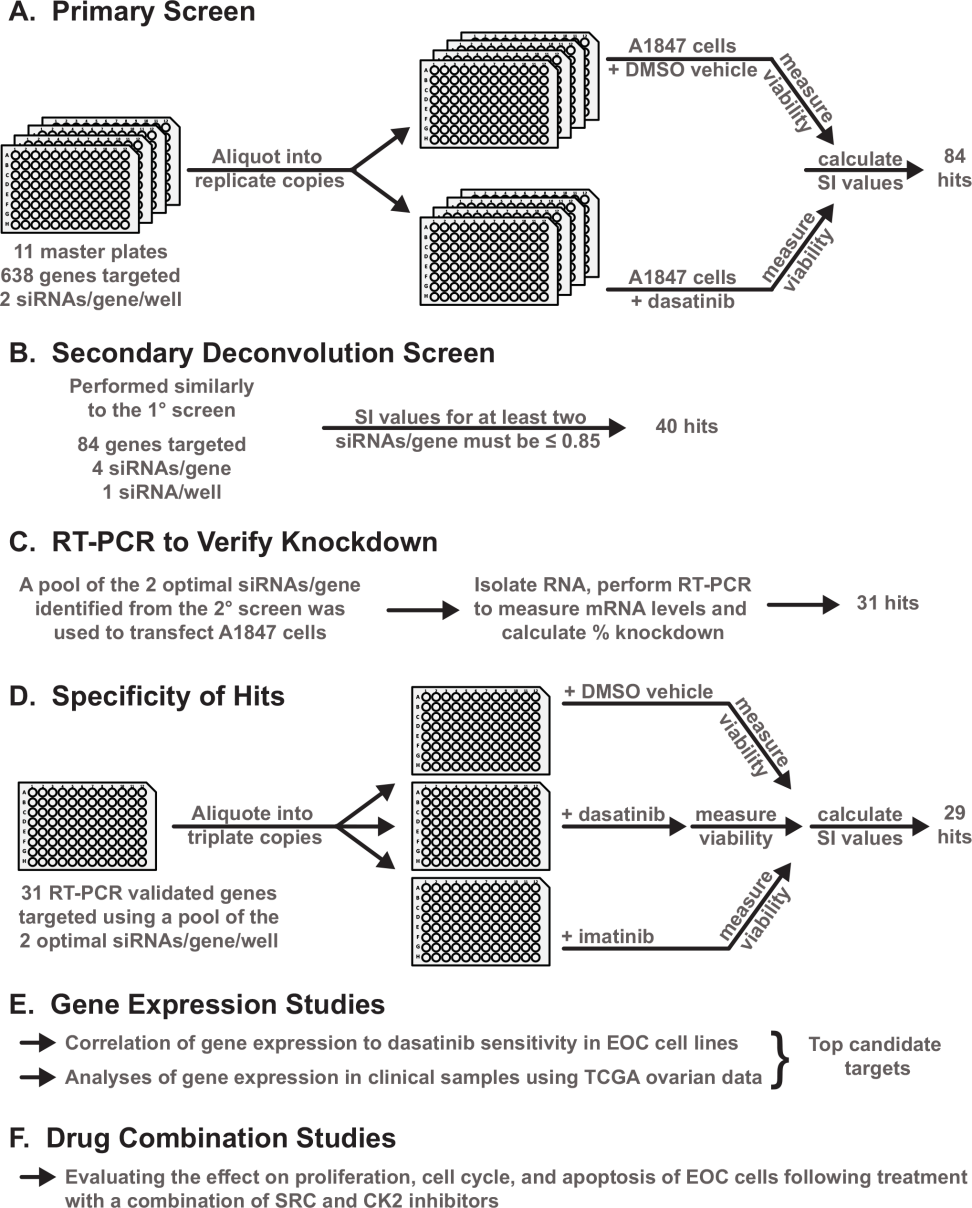
Overview of the design and work flow of experiments. **A**-**F.** A general schematic of the experimental workflow of the primary and secondary siRNA screening and subsequent validation and refinement experiments performed to identify the second-site sensitizers for dasatinib. Details for each set of experiments are provided in the subsequent Figures and Supplementary Figures and Tables throughout the Results section.

We used this focused library to screen an EOC cell line, A1847, derived from a patient with an untreated, undifferentiated ovarian carcinoma [36, 37], to identify genes that provide resistance to SFK inhibition via dasatinib treatment. This cell line was chosen because it provided consistent, reproducible transfection data under high-throughput screening conditions in a siRNA-based screen of the human druggable genome [38] and because it was sensitive to dasatinib relative to other EOC cell lines (Pathak and Godwin, unpublished results). We hypothesized that identification of second-site targets which further sensitize a sensitive cell line will certainly provide some degree of sensitization to more resistant cell lines. **Fig 1**provides a general schematic of the overall experimental design (see the Materials and Methods section for details of the screening). Following statistical analysis of the normalized cell viability data obtained from the primary screening of the A1847 cells in the presence of siRNA + dasatinib or siRNA + dimethyl sulfoxide (DMSO, the vehicle used to dissolve dasatinib), a sensitization index (SI) value was calculated for each siRNA pool. A total of 84 genes with an SI value ≤ 0.85 and a false discovery rate (FDR) < 10% representing ~13% of the genes targeted by this library were selected for inclusion in the next round of screening (**S1 Table**, primary screen).

### Eliminating false-positive hits

In order to eliminate potential false-positive hits from the initial screen that may be due to off-target effects of the initially used siRNAs, we assembled a new library containing four individual siRNAs (1 siRNA/well) targeting each of the 84 genes identified in the primary screen. With this new library of de-convoluted siRNAs, we performed a secondary screen in triplicate and calculated the SI value for each siRNA as was done for the primary screen. **S1 Fig** shows the correlation for 2 of the 3 screens for both the vehicle and dasatinib treatments. We considered genes as dasatinib-sensitizing only if they yielded an average SI value ≤ 0.85 for *at least* two of the four individual siRNAs targeting a particular gene across the three biological replicate experiments (**S1 Table**, secondary screen). Based on these more stringent criteria, we removed 44 potential false-positive hits identified in the initial screen.

For the remaining 40 hits, we pooled the two best siRNAs targeting each gene (*i*.*e*. those with the two lowest SI values identified from the secondary screens) and transfected the A1847 cells with this optimal pool of the two most effective siRNAs (2 siRNAs/well/gene) or the negative control siRNA, GL2, targeting the firefly-luciferase gene. Forty-eight hours following transfection, we performed quantitative RT-PCR to determine mRNA levels in cells targeted by the gene-specific siRNA pools relative to levels in cells targeted by the negative control siRNA. We considered as being on-target only those siRNA pools that reduced gene expression by at least 70%. Based on these criteria for the qRT-PCR results, we narrowed our hit list of dasatinib-sensitizing hits to 31 genes (**S2 Table**).

### Specificity of dasatinib-sensitizing hits

Given that dasatinib inhibits other tyrosine kinases in addition to the SFKs, such as BCR-ABL, c-KIT, and PDGFR [24], we next determined the level of specificity of the 31 hits for sensitizing EOC cells to SFK inhibition. We used the pool of the two best siRNAs targeting each gene (identified from the de-convolution studies) and screened the 31 hits in combination with dasatinib or in combination with another tyrosine kinase inhibitor, imatinib, which predominantly targets BCR-ABL, PDGFR, and cKIT, with limited activity against the SFKs [39, 40]. siRNA-mediated gene silencing in combination with imatinib resulted in only 2 of the 31 hits showing a sensitization effect of ≤ 0.85, whereas all 31 siRNA pools generated SI values ≤ 0.85 when screened in combination with dasatinib (**S3 Table**). The two hits identified from the imatinib combination screen (*GAB1* and *JUP*) were considered as being common sensitizers with dasatinib and therefore were removed from additional studies, refining the dasatinib-specific hit list further to 29 hits.

### Correlating gene expression to dasatinib sensitivity

**Fig 2:**
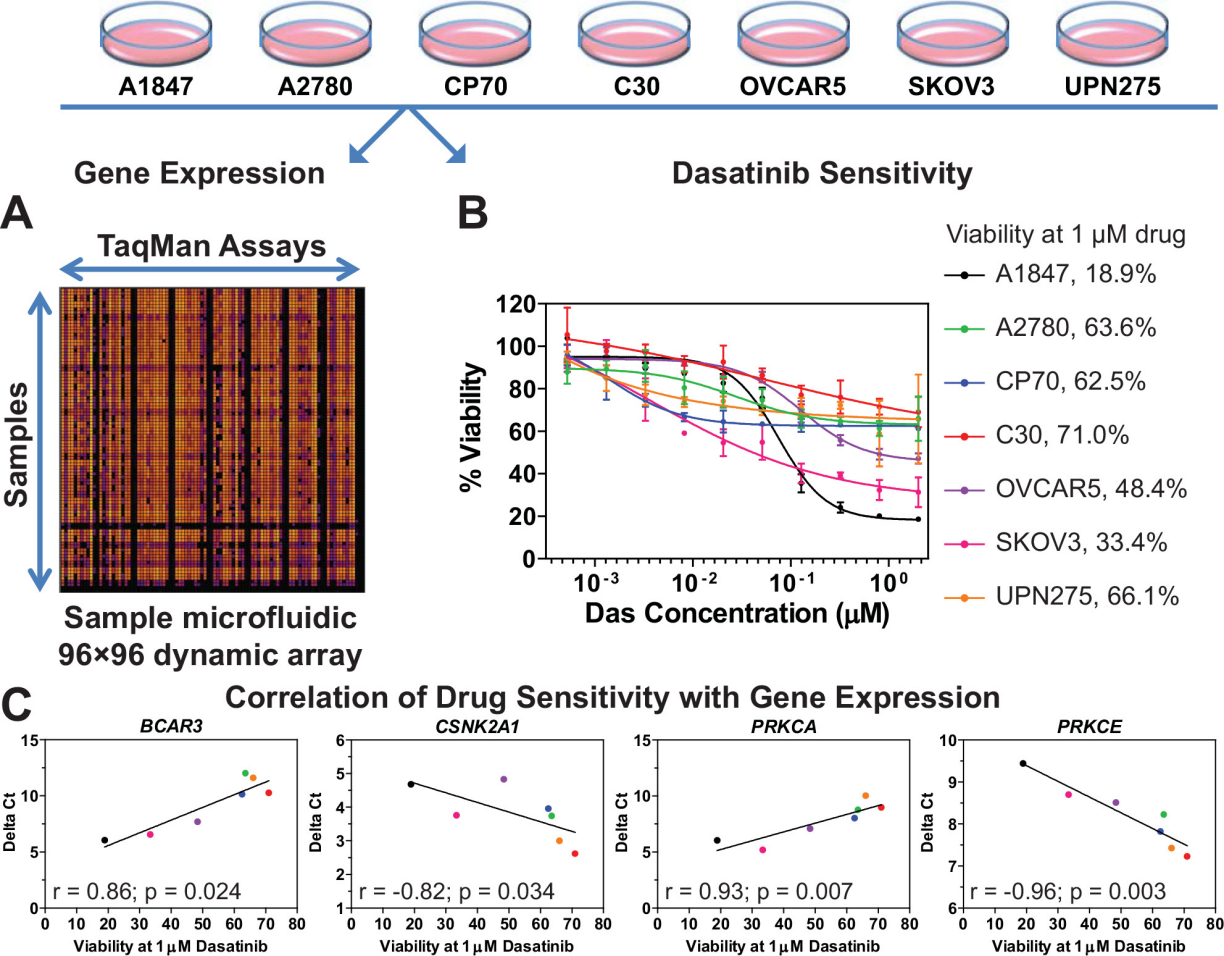
Correlation of gene expression to dasatinib sensitivity. **A.** The basal level of gene expression of 29 dasatinib-sensitizing genes in seven EOC cell lines was measured by using quantitative PCR performed with a 96☓96 dynamic array on the Fluidigm BioMark microfluidic platform. Shown is a representative heat map of the dynamic array. Delta C_t_ values were calculated for each gene in each cell line (see Materials and Methods for details). **B.** Data on the dose response to dasatinib for seven EOC cell lines were generated and cell viability at 1 μM dasatinib was calculated for each cell line as a percentage of vehicle treated cells using GraphPad Prism. Shown is the average ± standard error of mean for each data point. **C.** Delta C_t_ and dasatinib sensitivity data (i.e. viability at 1 μM drug concentration) were subjected to Spearman Correlation analysis using GraphPad Prism. The magnitude of correlation (Spearman r value) is shown for the four genes which showed a statistically significant correlation (p < 0.05). Each point represents an EOC cell line with the color matching the code shown in panel 2B. The line through the data points is for illustrative purposes only. **S3 Table** lists the r and p-values for the other genes evaluated but which did not show significance.

In order to further delineate the second-site sensitizers to dasatinib, the basal level of gene expression for the 29 dasatinib sensitizing hits was measured in seven EOC cell lines (A1847, A2780, C30, CP70, OVCAR5, SKOV3, and UPN275) using 96☓96 dynamic arrays on the BioMark microfluidic quantitative PCR (qPCR) platform (Fluidigm) (**Fig 2A**). These EOC cell lines were selected because they displayed varying levels of dasatinib sensitivity in previous unpublished work by Pathak and Godwin. Therefore, we obtained a more precise measurement of dasatinib sensitivity in these cell lines by measuring the dose response of each and calculating its viability at a dasatinib concentration of 1 μM (**Fig 2B**). The magnitude of correlation (Spearman coefficient r) and statistical significance (p-value) were calculated for each gene in order to identify potential genes whose expression levels correlated with dasatinib sensitivity (**S3 Table**). Four such genes (*BCAR3*, *CSNK2A1*, *PRKCA*, and *PRKCE*) were identified as being predictive of dasatinib sensitivity, with a statistically significant correlation between expression and drug sensitivity (p < 0.05, **S3 Table** and **Fig 2C**). Of these, *CSNK2A1* and *PRKCE* are inversely correlated with dasatinib sensitivity (*i*.*e*., low levels of these two genes result in higher sensitivity to dasatinib) and therefore are potential secondary targets (*e*.*g*., drugs which reduce their levels or activity in tumor cells would sensitize these cells to dasatinib).

### Clinical relevance of dasatinib sensitizers

**Fig 3:**
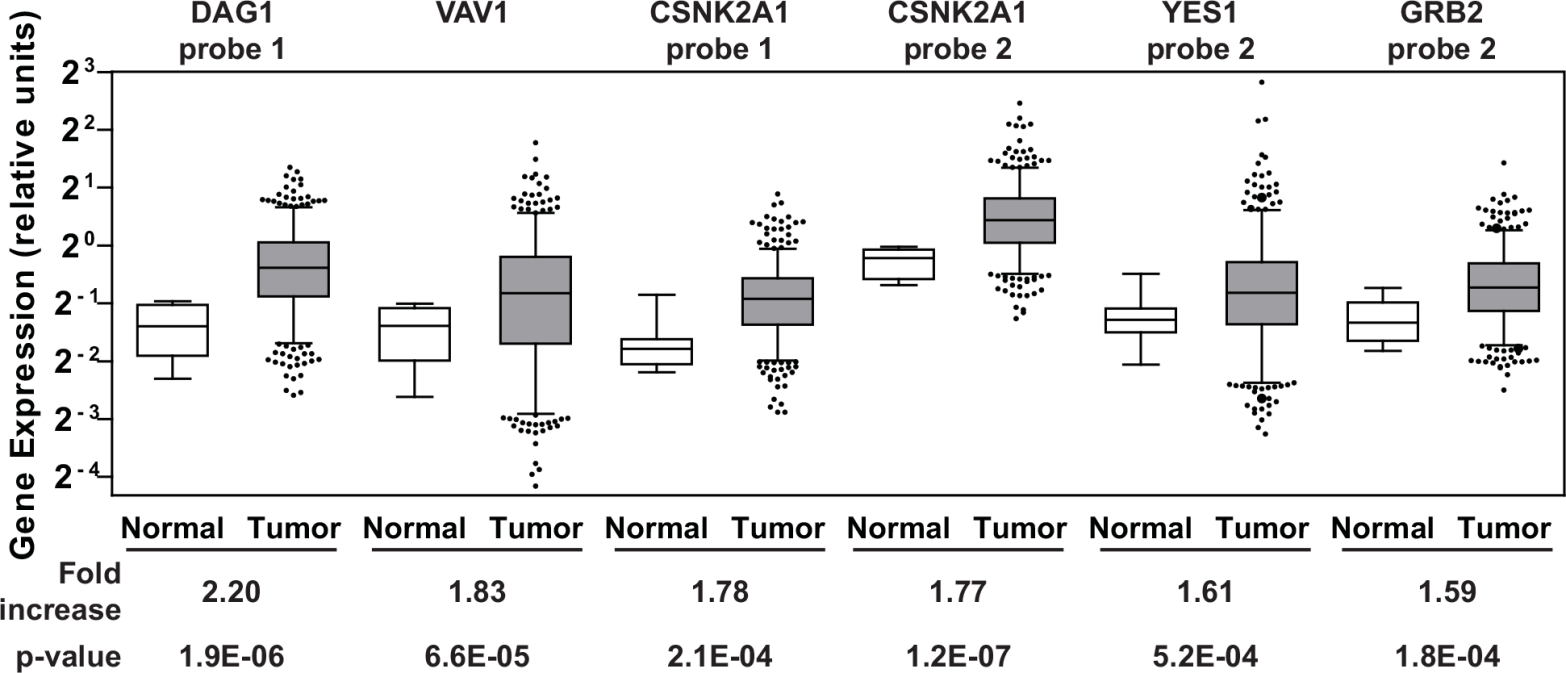
Gene expression in clinical samples. Agilent gene expression data from TCGA on 518 serous cystadenocarcinomas and 8 fallopian tube samples derived from healthy individuals were queried for 29 dasatinib sensitizing genes. The six Agilent probes that showed ≥ 1.5-fold increase in the average gene expression of the respective genes in the tumor samples (gray boxes) relative to the controls (white boxes) are shown. The whiskers of each box plot represent the expression values at the 5^th^ and the 95^th^ percentiles. The p-values were calculated using an unpaired two-tailed t-test using GraphPad Prism. **S4 Table** lists the average expression values of the Agilent probes across the tumor and normal samples for all 29 genes.

Genes whose protein products are aberrantly produced in excess in ovarian cancer patients can potentially be targeted with inhibitors to reduce their activity. These proteins would be considered clinically relevant therapeutic targets. Therefore, we analyzed gene expression data available through the Cancer Genome Atlas (TCGA) data portal on ovarian cancer [41] for the 29 dasatinib-specific sensitizers listed in **S3 Table**. Gene expression data from 518 serous cystadenocarcinomas, the major histological subtype for EOC, and fallopian tube samples from 8 healthy individuals were obtained from the database. The fallopian tube epithelium has been implicated as a source of origin for high grade serous ovarian cancer [42–45] and therefore an appropriate comparison group. Of the 29 genes that were surveyed, an average increase in gene expression levels greater than 1.5-fold in the tumor samples relative to the normal samples was observed in five genes (*CSNK2A1*, *DAG1*, *GRB2*, *VAV1*, and *YES1;* **Fig 3**and **S4 Table**). Interestingly, two of these genes (*CSNK2A1* and *DAG1*) were also among the top five hits with respect to the level of sensitization to dasatinib observed when the genes were silenced (**S3 Table**).

### *In vitro* drug combination of dasatinib and CX-4945

**Fig 4:**
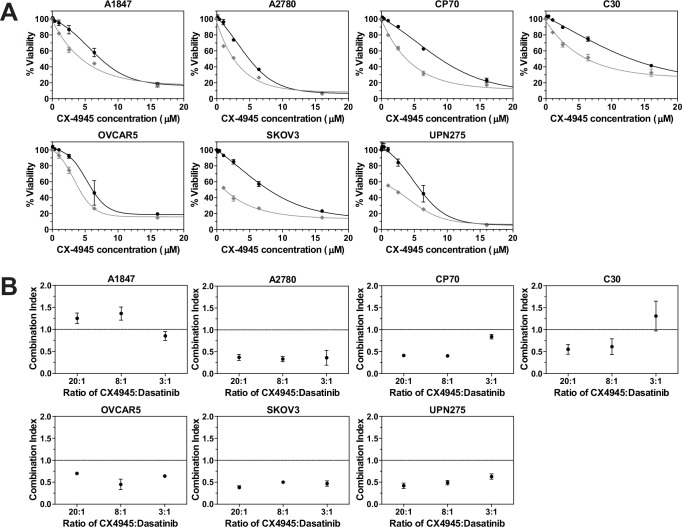
Drug combination using dasatinib and CX-4945. **A.** The Chou-Talalay method [78] was used to perform drug combination studies of dasatinib and CX-4945. The points represent the average viability ± standard error of mean following 72 h of drug treatment at the indicated concentrations of CX-4945 (•) and CX-4945 + dasatinib (◆; constant molar ratio of 20:1 of CX-4945:dasatinib) for the various EOC cell lines as a percentage of vehicle treated cells. The curve-fit lines were generated using non-linear regression analysis in GraphPad Prism. Data for the other molar ratios that were evaluated are presented in **S2 Fig. B.** The dose response data were used to calculate the Combination Index (CI) values for each cell line at the various molar ratios using CalcuSyn software [79]. CI values less than 1 suggest that the drugs are working synergistically. Shown is the average calculated CI value ± standard error of the mean.

Given its over-expression in a majority of the ovarian tumor samples, its low SI value from the screen, and its catalytic nature, *CSNK2A1* is a top candidate for potential drug screening/development and preclinical studies in combination with dasatinib. The *CSNK2A1* gene codes for the catalytic alpha subunit of protein kinase CK2, a serine-threonine kinase, and a CK2 inhibitor, CX-4945 (silmitasertib) [46–48], has recently completed a phase 1 clinical trial as a potential anticancer drug [32]. It is the only ATP-competitive inhibitor against CK2 to have this status. Therefore, we selected CX-4945 to study the effects on cell growth in a panel of established EOC cell lines when combined with dasatinib. Cells were treated with each single agent or a combination of the two drugs at a fixed molar ratio over a range of concentrations. **Fig 4A** shows the dose-response curves for CX-4945 as a single agent (black line) and when combined with dasatinib at a 20:1 molar ratio (gray dashed line). Dose response curves for two other molar ratios evaluated (8:1 and 3:1) are shown in **S2 Fig.** Combination Index (CI) values were calculated for each of the three ratios and are depicted in **Fig 4B**. Drug combinations which result in Chou-Talalay CI values less than 1 are considered to be synergistic whereas combinations which result in CI values greater than 1 are considered to be antagonistic [49, 50]. These data suggest that the drugs are working synergistically to inhibit proliferation across the majority of EOC cell lines.

**Fig 5:**
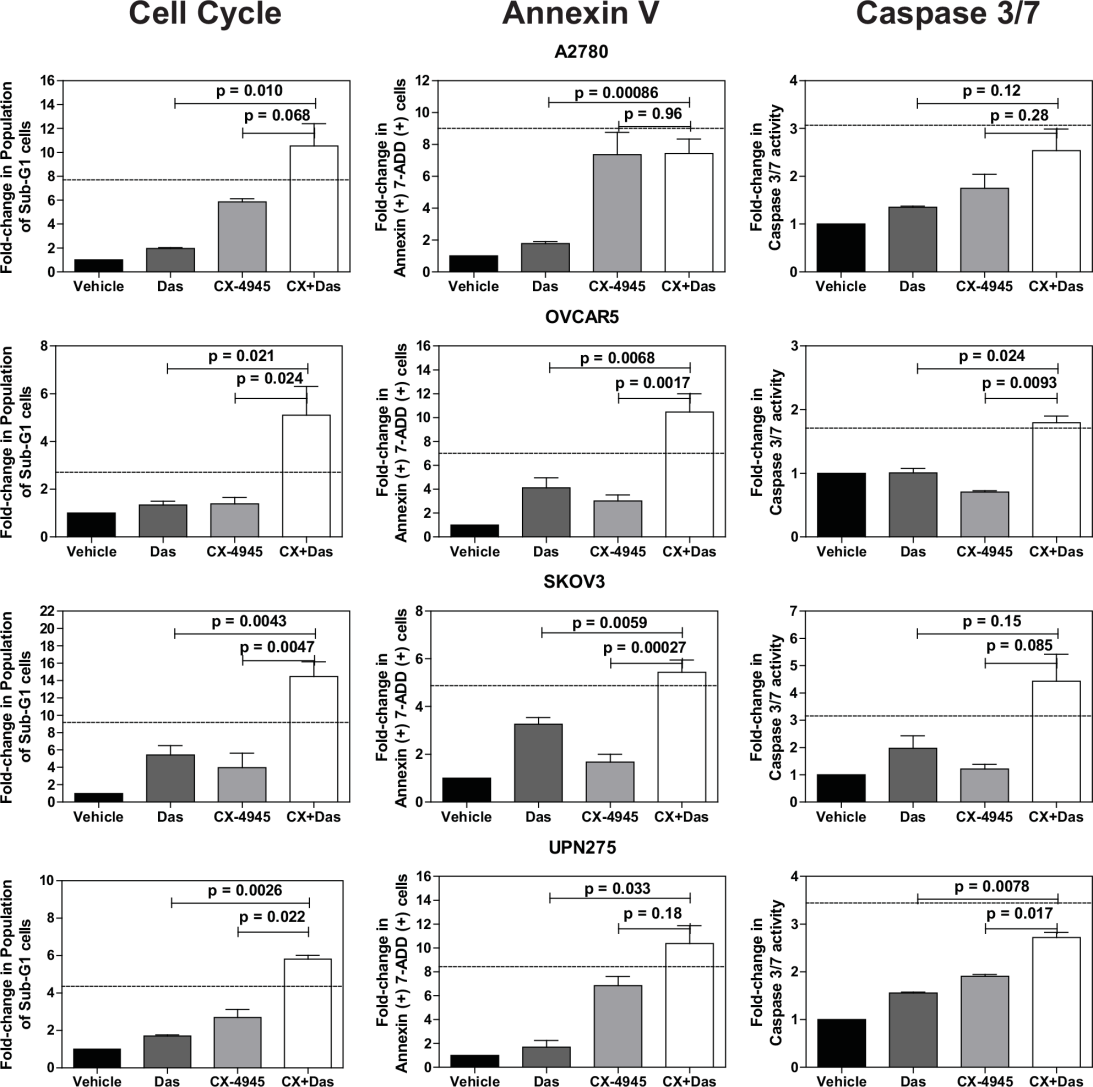
Quantification of cell cycle and apoptosis assays. Cell cycle and apoptosis data were quantified for the indicated fold-changes relative to vehicle treated cells and are presented as bar graphs showing the average fold-change ± standard error of mean. In all three assays, single (das, 0.5 μM; CX-4945, 10 μM) and combination drug treatments (das, 0.5 μM; CX4945, 10 μM) were for 72 h. P-values were calculated using a t-test comparing the combination treatment group to each single agent treatment group. The dashed line indicates the theoretical value if the drugs act additively calculated using the Bliss independence model (Bliss additivity value = FC_Das_ + (FC_CX-4945_ * (100—FC_Das_))/100 where FC is fold-change [51]. Observed values larger than the Bliss additivity value indicate synergy. See Materials and Methods for additional assay details.

We next evaluated the effects of the drug combination on cell cycle progression and cell death using propidium iodide staining (**S3 Fig**). We did not detect any significant changes in cell cycle phase distribution with either of the single agents at the doses used. However, the combination treatment increased the percentage of sub-G1 cells in the majority of the cell lines, suggesting the presence of cells undergoing apoptosis (**Fig 5**and **S3 Fig**). We therefore performed a direct measurement of apoptosis by measuring annexin V and cleavage of caspase-3 and -7 (**Fig 5**). Both assays indicated increased levels of apoptosis in the combination treated cells relative to the other treatments. In many instances, the increase in the sub-G1 and apoptotic cell population following combination treatment was greater than additive as indicated by values larger than those calculated using the Bliss independence model [51].

## Discussion

Dasatinib is an approved therapeutic for the treatment of several types of leukemia and is being used worldwide in patients both newly diagnosed and those previously treated for these diseases [26, 52–55]. It is also being investigated in early-stage clinical trials of other tumor types (http://www.clinicaltrials.gov/, [56]). Although, as we have previously reported, it showed a limited response as a single agent in previously treated patients with recurrent EOC [27], it is nonetheless a potent inhibitor of the SRC family and BCR-ABL tyrosine kinases that is well tolerated by patients and for which our studies provide preclinical rationale to evaluate in combination with other targeted agents. In addition, inhibition of the SFKs has been suggested as a mechanism to potentiate the anti-tumor activity of paclitaxel [57], the drug commonly used in front-line therapy for patients with EOC. Identifying ways to improve dasatinib activity against EOC would have significant and immediate clinical impact.

Towards this goal, we have taken a systematic, rational approach to identifying second-site molecular targets which sensitize EOC cells to dasatinib. We have identified five genes (*CSNK2A1*, *DAG1*, *GRB2*, *PRKCE* and *VAV1*) as candidate second-site sensitizers to dasatinib and four genes (*BCAR3*, *CSNK2A1*, *PRKCA*, and *PRKCE*) as predictors of dasatinib sensitivity. Although *YES1* was identified as a potential second-site sensitizer (**Fig 3**and **S4 Table**), we did not consider it as a viable option for combination with dasatinib. This is because *YES1* codes for YES, a member of the SFK with a high degree of structural and functional homology with other family members [58, 59]. In addition, dasatinib inhibits YES with the same selectivity as other SFK members [24]. Therefore, we do not consider *YES1* as a second-site molecular target given that it appears dasatinib directly inhibits YES1 already. Of the remaining candidates, *CSNK2A1* is the top candidate for combination therapy based on the results presented in this study. Its role in the biology of the cell and ovarian cancer and its interactions with SFK members is briefly discussed below. A similar discussion is provided for the remaining hits as **Supporting Information**.

*CSNK2A1* codes for the catalytic alpha subunit of protein kinase CK2. This protein is a ubiquitous, constitutively active serine/threonine kinase with essential roles in cell viability and division, and in suppression of apoptosis [60–62]. CK2 is a promiscuous kinase, with over 300 potential substrates [63] and a dual role both in cell proliferation and death makes, that has been implicated in many diseases [61]. CK2 protein levels are elevated in all cancers that have been examined [62, 64]. TCGA data on ovarian cancer shows that the catalytic alpha subunit of CK2 is overexpressed in a majority of ovarian tumors (**Fig 3**) and the MEK-MAPK-CK2 pathway has been implicated in phenotypic changes of a cell culture model representing progressive stages in the development of ovarian cancer [65]. CK2 has been identified as a promising therapeutic target for cancer therapy [62] and CX-4945, the clinical stage CK2 inhibitor which has been evaluated in phase 1 trials, was shown to synergize with gemcitabine and platinum-based chemotherapeutics *in vitro* using ovarian cancer cell lines and *in vivo* using a mouse xenograft model of ovarian cancer [51]. CK2’s relationship with the SFK members is enigmatic with studies showing that CK2 can down-regulate the activity of at least three SFK members (SRC, FYN, and YES) via direct phosphorylation of their threonine residues [66, 67] while another study shows that tyrosine phosphorylation of CK2 by SFK members increases CK2 catalytic activity [68]. How these two kinases interact with one another and whether this is direct or through molecular chaperones such as CDC37 [69] or other signaling pathways and how their activities are modulated is not entirely clear at the moment. Detailed mechanistic studies are needed to provide a more thorough picture and to help generate a rationale for how synergy between these two kinases may occur. Nevertheless, additional preclinical development using dasatinib and CX-4945 or other relevant inhibitors targeting SFK and CK2 for the treatment of epithelial ovarian cancer are warranted.

Given the time and money invested and the most precious resources of all, the patients, into developing safe, highly potent targeted drugs such as dasatinib and other similar molecularly targeted agents, it is imperative that all potential avenues to improve these existing drugs be evaluated. This includes identifying rational drug combinations, reformulation for alternate routes of drug delivery, and repurposing for other diseases for which the drug was not initially approved. Finally, identification of predictive biomarkers of response is essential for targeted therapies in order to have greater precision in patient selection so that a true response rate is measured for a given drug without being complicated by inclusion of patients who do not respond simply because they were not suitable candidates to benefit from a particular drug. In this study, we have taken a focused approach to identify several candidates for combination therapy with dasatinib, a strategy that can be applied to other targeted agents that have similarly shown poor clinical activity.

## Materials and Methods

### Cell culture

Epithelial ovarian cancer cell lines used in this study have been previously established or derived by us under a protocol approved by the Fox Chase Cancer Center Institutional Review Board and written informed consent (C30 and CP70 from parental A2780 cells [70]; UPN275 [38]). Cell lines established by other institutions were obtained as gifts from Dr. Thomas Hamilton while at the Fox Chase Cancer Center (Philadelphia, PA) (A1847 and A2780 [36]; OVCAR5 [71]; SKOV3 [72]). All of the cell lines were maintained in normal growth media consisting of RPMI 1640 media supplemented with fetal bovine serum (10% (vol/vol)), insulin (7.5 μg/mL), penicillin (100 U/mL), and streptomycin (100 μg/mL) at 37°C in a humidified atmosphere with 5% CO_2_.

### siRNA screening

Details on the design and assembly of the siRNA library used in the primary screen were previously described by Astsaturov *et al*. [33]. Briefly, the library was assembled using siRNAs from Qiagen and consisted of eleven 96-well plates containing two siRNAs duplexes per well targeting a total of 638 genes arrayed into the inner 60 wells of each plate. The siRNA library used in the secondary deconvolution screening was assembled using siRNAs from Qiagen and consisted of four individual siRNA duplexes for each of the genes targeted in the deconvolution screens arrayed one siRNA duplex per well into the inner 60 wells of 96-well plates. For both the primary and secondary screens, positive control siRNAs targeting polo-like kinase 1 (*PLK1*) and negative control siRNAs targeting the firefly luciferase gene (GL2) were included in quadruplicate on every plate. All siRNA transfections were done with a reverse transfection method [73] using DharmaFECT-1 (Dharmacon) as the cationic lipid transfection reagent. For the primary screen, DharmaFECT-1 was diluted in reduced-serum media (OptiMEM, Invitrogen) and added to the siRNAs arrayed in v-bottom 96-well dilution plates using a bulk reagent microplate dispenser. The siRNA-lipid complexes were allowed to form for 30 min at room temperature. Each siRNA-lipid complex was then aliquoted equally into four 96-well flat-bottom test plates using a CyBio Vario liquid handler followed by addition of A1847 cells in normal growth media lacking antibiotics using a bulk reagent microplate dispenser (6,500 cells/well, ∼95 μL final volume/well). The final DharmaFECT-1 dilution after cells were seeded was 1:700 and the final concentration of the siRNA duplex pools was ~50 nM (~25 nM of each individual siRNA duplex). After 24 h, two of the four copies from each set of plates were treated with a sub-lethal dose of dasatinib (LC Labs; 5 μL/well, ~30 nM final, IC_10-15_) and the other two copies were treated with an equal volume of the vehicle used to prepare the drug such that the final DMSO concentration was the same (0.85% DMSO). Seventy-two hours following drug addition, cell viability was determined by using CellTiter-Blue (CTB) (Promega). The CTB reagent was diluted 3-fold in Ca^2+^/Mg^2+^-free Dulbecco’s phosphate buffered saline (PBS) (1 part CTB + 2 parts PBS) prior to its addition to the assay plates (20 μL per well added using a bulk reagent microplate dispenser). Fluorescence intensity (FI) was measured by using the Envision (Perkin Elmer) multi-label microplate reader 3 h following addition of the CTB reagent. Secondary deconvolution screens were performed using this same protocol with a final siRNA concentration of 25 nM for the siRNA duplex in each well. When imatinib was included in the screening procedure, a final concentration of 800 nM (IC_10-15_ for A1847 cells) was used. FI data from all screens were analyzed as described below.

### Statistical analysis for siRNA screens

To calculate cell viability following siRNA treatment, the FI value from each well targeted by gene-specific siRNAs was divided by the mean FI value from four reference wells containing the non-targeting negative control GL2 siRNA on each plate to yield a viability score (V) defined as V = (fluorescence intensity_specific siRNA_)/(mean fluorescence intensity_GL2 siRNA_)) corresponding to each gene. The sensitization index (SI) of each siRNA was then defined as the viability of cells in the presence of siRNA and drug divided by the viability of the cells in the presence of siRNA and vehicle (SI = (V_siRNA + drug_)/(V_siRNA + vehicle_)). All calculations were automated using the cellHTS2 package [74] within the Bioconductor open source software package (http://bioconductor.org) [75]. The effect of drug treatment on viability was measured based on the normalized viabilities in the drug treated and vehicle wells using Limma [76]. Limma borrows strength across genes based on an empirical Bayes approach and identifies statistically significant changes in viability by combining information from a set of gene-specific tests. Hits were identified based on statistical significance as well as biological significance. Statistical significance was determined by p-value controlled for the false discovery rate (FDR) using the Benjamini-Hochberg method [77] to account for multiple testing. Hits showing an FDR of less than 20% were considered statistically significant. Biological significance was arbitrarily defined as a decrease in the SI greater than 15%. Hits identified as statistically and biologically significant were further validated.

### Quantitative RT-PCR to measure gene knockdown

Forty-eight hours following transfection, total RNA was extracted from 4 replicate wells for each siRNA pool using TRIzol reagent (Invitrogen). Each well was independently transfected with the siRNA pool. The extracted RNA from the 4 wells was pooled and quantified using a Nanodrop 1000 instrument (Thermo Scientific). Approximately 1 μg of the pooled total RNA was reverse transcribed to cDNA in a 20 μL reaction containing 100 ng of random hexamers, 500 μmol/L of deoxynucleoside triphosphate mix, 10 mM dithiothreitol (DTT), 20 units of M-MLV reverse transcriptase (SuperScript II), and 1☓ First Strand reverse transcription buffer (all reagents for reverse transcription were from Invitrogen). Quantitative PCR (qPCR) reactions (20 μL) were assembled in 384-well plates in triplicate wells consisting of 50 ng of cDNA, 1☓ TaqMan Gene Expression Assays (Applied Biosystems), and 1☓ TaqMan Universal PCR Master Mix (Applied Biosystems). PCR product was detected in real time using an ABI7900 instrument (Applied Biosystems). Relative mRNA levels for each gene were assessed following normalization to the internal reference control, cyclophilin A (*PPIA*) mRNA levels. The cycle threshold (Ct) values were determined from the amplification curves for each sample and then normalized to the level of *PPIA* in that sample to calculate a ΔCt for each siRNA treated sample as follows: ΔCt_gene of interest or GL2 siRNA treated_ = Ct_gene of interest or GL2 siRNA treated_—Ct_PPIA_. The amount of mRNA remaining following siRNA treatment was calculated as follows: 2^-ΔΔCt^, where ΔΔCt = ΔCt_gene of interest siRNA treated_ - ΔCt_GL2 siRNA treated_. The percent of gene knockdown following siRNA treatment was calculated as follows: (1-2^-ΔΔCt^)*100.

### Correlation of gene expression and drug sensitivity

The Fluidigm BioMark microfluidic qPCR platform was used to run 96☓96 dynamic arrays to measure the basal mRNA expression levels in EOC cell lines grown as sub-confluent monolayer cultures. Total RNA from sub-confluent monolayer cultures was extracted using TRIzol, quantified, and 200 ng of total RNA was reverse transcribed as described above. The resulting cDNA for each sample was then pre-amplified using a multiplexed specific target amplification protocol (Applied Biosystems) where each gene-specific TaqMan assay was pooled and used to perform 14 cycles of a pre-amplification reaction with each gene-specific TaqMan assay at a final concentration of 0.05x. The resulting pre-amplified cDNA was then diluted 5-fold and used as the input cDNA for the subsequent qPCR using the 96☓96 dymanic arrays on the BioMark platform following manufacturer recommended protocols. For each cell line, two biological replicate samples were obtained via two independent RNA extractions, reverse transcription reactions, and qPCRs; six technical replicates for each biological replicate were included on the dynamic arrays. The average ΔCt value from all biological and technical replicates for each gene in each sample was calculated as described above using *PPIA* as the housekeeping gene. The average ΔCt value for each gene from each EOC cell line and the cell viability data of all seven EOC cell lines at 1 μM dasatinib (measured as described in the **Supplementary Information** section) were subjected to Spearman Correlation using GraphPad Prism to calculate the direction and magnitude of correlation (Spearman r value) and statistical significance between the basal level of gene expression of a given gene and sensitivity to dasatinib across these cell lines.

### Dasatinib drug sensitivity assays

After cell enumeration, EOC cell lines were seeded into 96-well plates (2 x 10^3^ cells/well) using normal growth media described above for the EOC cells. Twenty-four hours following seeding, a 2.5-fold serial dilution of dasatinib was freshly prepared in DMSO/media and added to the cells in triplicate wells maintaining a final DMSO concentration of 0.25% in all vehicle and drug treated wells. Seventy-two hours following drug addition, 20 μL of diluted CTB was added to each well to measure cell viability as described above. Two hours following CTB addition, FI was measured by using an Infinite M200 PRO microplate reader (Tecan). Viability was defined as a percentage of the ratio of FI values from drug treated wells to vehicle treated wells. Assays were performed minimally as biological duplicates using triplicates wells within each experiment. GraphPad Prism was used to fit the average values of the dose-response data to a four-parameter equation (Y = bottom + (top—bottom)/(1+10^(LogIC50—X)*HillSlope^)). Cell viability at 1 μM dasatinib concentration was interpolated from the curve fit.

### Drug combination assays

Drug combination studies were performed using the combination index (CI) method described by Chou and Talalay [78]. Briefly, EOC cells were seeded into 96-well plates (2 x 10^3^ cells/well) as described above. Twenty-four hours after seeding, serial dilutions of either dasatinib, CX-4945, or both dasatinib and CX-4945 were freshly prepared in DMSO/media and added to the wells either as a single agent or as a combination such that the two drugs were always at a constant molar ratio in each serial dilution. Assays were performed as biological duplicates using triplicate wells within each experiment. Cell viability following 72 h of treatment was evaluated using CTB as described above and the viability data were then analyzed using CalcuSyn (ver 2.1, BioSoft, UK) [79] to calculate the synergy between the two drugs at each molar ratio evaluated. Drug combinations which yielded CI values less than 1 were considered to be synergistic [49, 50].

### Cell cycle and apoptosis analysis

EOC cell lines were seeded in 6 cm dishes (2 x 10^5^ cells/dish) and synchronized overnight in the presence of 2 mM thymidine. Three hours after removal of the thymidine block, the cells were treated with either vehicle (0.25% DMSO), dasatinib (0.5 μM), CX-4945 (10 μM), or the combination of both drugs (das, 0.5 μM; CX4945, 10 μM). Seventy-two hours after treatment, floating and adherent cells were collected by trypsin treatment. A portion of the cells were immediately fixed using 70% ice-cold ethanol and stored overnight at -20°C before being used for cell cycle analysis using propidium iodide staining (Guava Cell Cycle reagent, EMD Millipore) following the manufacturer’s established protocol. The remaining cells were used to perform apoptosis analysis using the Guava Nexin reagent (EMD Millipore) which contains a premixed cocktail of phycoerythrin-conjugated Annexin V and a cell impermeant dye (7-AAD) following the manufacturer’s established protocol. The cell cycle assays were performed three independent times with two technical replicates for each. The apoptosis assays were performed four independent times with two technical replicates for each. A Guava Easycyte HT instrument (EMD Millipore) was used to measure the changes in cell cycle and apoptosis levels.

### Caspase 3/7 measurement

EOC cells were seeded into 96-well plates (2 x 10^3^ cells/well) as described above. Twenty-four hours after seeding, cells were treated with vehicle (0.25% DMSO), dasatinib (0.5 μM), CX-4945 (10 μM), or both drugs (das, 0.5 μM; CX4945, 10 μM). Following 72 h of treatment, cells were subjected to Caspase-3 and -7 activity measurement using the Caspase-Glo 3/7 assay (Promega) following the manufacturer’s protocol. Luminescence, which was directly proportional to caspase activity, was measured using the Tecan Infinite M200 PRO microplate reader. The caspase 3/7 measurements were performed two independent times with two technical replicates for each.

### Analysis of TCGA data

The log_2_ transformed Agilent gene expression values for the 29 genes of interest were downloaded from the ovarian cancer data portal supplied by TCGA (http://tcga-data.nci.nih.gov/tcga/tcgaHome2.jsp) on 518 serous cystadenocarcinomas and 8 organ-specific healthy control samples. Anti-log values were derived and the mean expression values for tumor samples and control samples were calculated. The fold-change in the average expression in the tumors relative to the average expression in the controls was calculated and a Student’s two-tailed t-test was performed to calculate a probability value, p. A fold-change of ≥ 1.5 with an associated p-value < 0.05 was considered as a significant difference in expression between the tumor and control samples.

## Supporting Information

S1 Fig Correlation between replicate siRNA screens. Viability scores from two independent biological replicate experiments evaluating 336 siRNAs from the secondary deconvolution siRNA screens using **A.** siRNA + vehicle or **B.** siRNA + dasatinib were subjected to Spearman Correlation analysis using GraphPad Prism. The magnitude of correlation (Spearman r value) and the statistical significance of the correlation of the viability scores are shown. These 336 siRNAs are targeting the 84 genes (4 siRNA/gene; 1 siRNA/well) identified in the primary screen (**S1 Table**).
(EPS)

S2 Fig Combination of SFK and CK2 inhibitors at two additional molar ratios. The Chou-Talalay method was used to perform drug combination studies of dasatinib and CX-4945. The points represent the average viability ± standard error of mean following 72 h of drug treatment at the indicated concentrations of CX-4945 (•) and CX-4945 + dasatinib (◆; constant molar ratio of 20:1 of CX-4945:dasatinib) for the various EOC cell lines as a percentage of vehicle treated cells. The curve-fit lines were generated using non-linear regression analysis in GraphPad Prism. **A.** Shown are the data for a constant molar ratio of 8:1 of CX-4945:dasatinib and **B.** 3:1 of CX-4945:dasatinib.
(EPS)

S3 Fig Cell cycle phase distribution. EOC cell lines were treated with vehicle or the indicated single agents (das, 0.5 μM; CX-4945, 10 μM) or combination of drugs (das, 0.5 μM + CX4945, 10 μM) for 72 h. Cells were harvested, ethanol-fixed, and analyzed by using propidium iodide. Shown are representative histograms of the various phases of the cell cycle for the indicated EOC cell lines following drug treatment. These data were used to calculate the percentage of cells in the sub-G1 phase relative to vehicle treatment to generate the bar graphs in **Fig 5**.
(EPS)

S1 Table Primary Screen – 84 genes out of 638 targeted genes were selected as "hits" following the primary screen using pooled siRNAs (2 per well). Secondary Screen–Forty hits were validated following secondary screening using individual siRNAs (1 per well; 4 per gene). The two siRNAs identified as being optimal for each gene are indicated and were selected for use in additional studies.
(PDF)

S2 Table The percent reduction in gene expression following siRNA treatment using a pool of the two optimal siRNAs per gene identified from the secondary deconvolution screens is shown. The 31 genes selected for additional studies are indicated.
(PDF)

S3 Table Specificity of hits – Sensitization index values were calculated following treatment with dasatinib or imatinib. The two genes highlighted in yellow appear to synergize with both drugs (SI ≤ 0.85) and therefore we did not use them for further studies. The remaining 29 hits were considered as dasatinib-specific sensitizers. Correlation of hits–The Spearman coefficient (r) and statistical significance (two-tailed t-test) for 29 genes were calculated using GraphPad Prism to determine the correlation of gene expression with dasatinib sensitivity. Genes highlighted in green show statistically significant correlation of basal gene expression with dasatinib sensitivity. ND, not determined.
(PDF)

S4 Table An analysis of ovarian TCGA Agilent gene expression data of the 29 dasatinib sensitizing hits in 518 serous cystadenocarcinomas and 8 fallopian tube samples derived from healthy individuals was performed. Shown are the hits ranked by the average fold-change in gene expression in the tumor samples relative to the control tissue. Data from multiple probes are shown when available.
(PDF)
