# Supplementary material for: A Synthetic Lethality Screen Using a Focused siRNA Library to Identify Sensitizers to Dasatinib Therapy for the Treatment of Epithelial Ovarian Cancer
Source: PLoS One. 2015 Dec 4;10(12):e0144126. doi: 10.1371/journal.pone.0144126 (PMC4670180; doi:10.1371/journal.pone.0144126)
Supplement: S1 Table — Secondary Screen–Forty hits were validated following secondary screening using individual siRNAs (1 per well; 4 per gene). The two siRNAs identified as being optimal for each gene are indicated and were selected for use in additional studies. (PDF) [file pone.0144126.s004.pdf]

S1 Table

Primary Screen					Secondary Screen					
	Gene ID	Gene Symbol	SI Value from the Primary Screen	siRNAs Included in the Primary Screen	siRNAs Used in the Secondary Screen	Qiagen siRNA ID	siRNATarget Sequence	Average SI Value from Secondary Screens (n = 3)	Standard Deviation	siRNAs Selected as Hits from the Secondary Screen
1	25	ABL1	0.66		Hs_ABL1_10	SI00288316	ACGCACGGACATCACCATGAA	0.84	0.01	
					Hs_ABL1_11	SI00288323	CCAGTGGAGATAACACTCTAA	0.60	0.04	✓
				✓	Hs_ABL1_8	SI00299103	AACCAAGCCTTTGAAACAATG	0.97	0.04	
				✓	Hs_ABL1_9	SI00299110	AACGGCTGATGTGGACTGTCT	0.75	0.10	✓
2	309	ANXA6	0.76		Hs_ANXA6_5	SI04161528	AAGGGACTCGGGACTGACGAA	1.01	0.01	
					Hs_ANXA6_6	SI04200336	TGGCCGGGACTTAATGACTGA	0.77	0.01	✓
					Hs_ANXA6_7	SI04270917	TCGGTTGGTGTTCGATGAGTA	0.83	0.03	✓
					Hs_ANXA6_8	SI04339608	CGGGCAAGTTTGAACGGTTGA	0.86	0.06	
3	50807	ASAP1	0.81		Hs_DDEF1_5	SI04135145	CAGACTCGCCCACATCACCAA	0.90	0.06	
					Hs_DDEF1_6	SI04181800	GAGGCGAGTGAAGACCATTTA	1.01	0.01	
					Hs_DDEF1_7	SI04309088	CACTGTGGATATAGTTAACCA	1.01	0.02	
					Hs_DDEF1_8	SI04341750	CCCGCCCGAAATCTTTCAGAA	1.05	0.02	
4	430	ASCL2	0.85		Hs_ASCL2_2	SI00073220	AGGGAGCGTGAACTTTATAAA	0.97	0.01	
					Hs_ASCL2_3	SI00073227	CACGCTTTGGGCCTTACAGAA	0.94	0.05	
					Hs_ASCL2_4	SI00073234	AAGGAAGGAGGGCGTGAGAAA	0.92	0.04	
				✓	Hs_ASCL2_6	SI03091018	CTCGACTTCTCCAGCTGGTTA	0.63	0.02	
5	56751	BARHL1	0.73	✓	Hs_BARHL1_3	SI00310331	CAGGACTAAATGGAAGCGACA	0.96	0.05	
					Hs_BARHL1_5	SI04130868	CACGGCCTTCACCGACCATCA	0.94	0.10	
					Hs_BARHL1_6	SI04222771	CTCATCGGACTCTGAGTATAA	0.95	0.03	
					Hs_BARHL1_7	SI04238710	GCGGATGTTCCCGTCGCCTTA	0.96	0.04	
6	8412	BCAR3	0.83		Hs_BCAR3_2	SI00053095	AAGGTATCAGTTATATGATAT	1.01	0.02	
					Hs_BCAR3_3	SI00053102	GCCCAACGAGTTTGAGTCAAA	0.73	0.10	✓
				✓	Hs_BCAR3_5	SI03080196	CCGAGCGGCCACTCTGAGTAA	0.77	0.13	✓
				✓	Hs_BCAR3_6	SI03081603	CCGGAACTCTGGCGTCAACTA	1.04	0.06	
7	602	BCL3	0.76		Hs_BCL3_2	SI00073290	CACGATGTAAATTATTAAGCA	0.85	0.03	
					Hs_BCL3_3	SI00073297	CTCGACATCTACAACAACCTA	0.87	0.04	
				✓	Hs_BCL3_5	SI02654554	CAACGTGAACGCGCAAATGTA	0.55	0.02	✓
				✓	Hs_BCL3_6	SI03082156	CCGGCCGGAGGCGCTTTACTA	0.68	0.00	✓
8	54876	C4orf30	0.76		Hs_C4orf30_1	SI04162718	CGGGAGATCTGCCGAGGATAA	1.08	0.08	
					Hs_C4orf30_2	SI04163901	TCTGACTAAATCAGCCTATAA	0.96	0.01	
				✓	Hs_C4orf30_3	SI04284903	CCAGATGTTCTTGACAGCTCA	1.03	0.01	
				✓	Hs_C4orf30_4	SI04373012	ATGGGCCACAAGCATACTTTA	0.70	0.05	
9	805	CALM2	0.65		Hs_CALM2_3	SI00027566	TTGTTTGTAACTTATCTGTAA	0.83	0.07	✓
					Hs_CALM2_5	SI00605332	ACAAAGGAATTGGAACTGTA	0.98	0.09	
				✓	Hs_CALM2_8	SI02758413	GACCTTGTACAGAATGTGTTA	1.08	0.04	
				✓	Hs_CALM2_9	SI02758420	AAGCCCTTCTGCACATCTAAA	0.73	0.04	✓
10	868	CBLB	0.83		Hs_CBLB_1	SI00156765	TCGGTTGGCAAACGTCCGAAA	0.94	0.04	
					Hs_CBLB_2	SI00156772	TCCGGTTAAGTTGCACTCGAT	1.02	0.01	
				✓	Hs_CBLB_3	SI00156779	ACGGGCAATAAGACTCTTTAA	0.72	0.07	
				✓	Hs_CBLB_5	SI03072398	CAGGTGTTGCAGCATCATTGA	0.87	0.03	
11	896	CCND3	0.78		Hs_CCND3_1	SI00027860	GACCATCATCCTACTGTAATA	1.02	0.04	
					Hs_CCND3_2	SI00027867	CAGGAACCACACCACATCTAA	0.63	0.02	✓
				✓	Hs_CCND3_4	SI00027881	TGGGACAGAATTGGATACATA	0.82	0.01	
				✓	Hs_CCND3_5	SI03073924	CATGCGGAAGATGCTGGCTTA	0.75	0.02	✓
12	919	CD247	0.83		Hs_CD3Z_1	SI00014441	TTGGTTATATTTAGCTCCAAA	0.99	0.06	
					Hs_CD3Z_2	SI00014448	CACGCTTTCAGCGAATGACAA	0.76	0.04	✓
				✓	Hs_CD3Z_4	SI00014462	CAGGAAGGCCTGTACAATGAA	0.70	0.03	✓
				✓	Hs_CD247_1	SI03029397	AACGAGCTCAATCTAGGACGA	0.87	0.02	
13	916	CD3E	0.82		Hs_CD3E_1	SI00014413	CTCCGCCATCTTAGTAAAGTA	0.95	0.06	
					Hs_CD3E_5	SI02624230	CAGGATATTTATTTGTGCTAT	0.78	0.03	✓
				✓	Hs_CD3E_6	SI03055598	CACAATTGTCATAGTGGACAT	0.78	0.04	
				✓	Hs_CD3E_7	SI03088848	CTCAGTATCCTGGATCTGAAA	0.75	0.03	✓
14	1001	CDH3	0.71	✓	Hs_CDH3_5	SI02663934	CAGATGAAATCGGCAACTTTA	0.59	0.02	
				✓	Hs_CDH3_6	SI02663941	AAGCCTCTTACCTGCCGTAAA	0.98	0.02	
					Hs_CDH3_10	SI04434633	AAGGGTGAGGACAATCGTGTA	1.04	0.02	
					Hs_CDH3_11	SI04434640	CACCCGTGACAACGTCTTCTA	1.05	0.00	
15	10153	CEBPZ	0.75	✓	Hs_CEBPZ_6	SI03178007	CAGTACCACACCGAAAGTAAA	0.97	0.07	
				✓	Hs_CEBPZ_7	SI03237710	TGGATCGATTTGTATACCGAA	0.79	0.01	
					Hs_CEBPZ_8	SI04150965	CAGGGTTGATGACGTGTTCCA	1.00	0.04	
					Hs_CEBPZ_9	SI04337977	AAGGCTACTCTTCCGCTCAAA	1.10	0.04	
16	1398	CRK	0.75	✓	Hs_CRK_1	SI00073780	CAGGATGTACCGAGCACTTTA	0.71	0.05	✓
					Hs_CRK_3	SI00073794	CTGAGTATAGTTCAACAGTTT	0.99	0.05	
				✓	Hs_CRK_5	SI00299929	AATCCGGGACAAGCCTGAAGA	0.85	0.06	✓
					Hs_CRK_6	SI03105200	GCGAGCCCTCTTTGACTTTAA	0.92	0.04	
17	1439	CSF2RB	0.81		Hs_CSF2RB_1	SI00008659	CTCCTTTGGCCTATTCTACAA	0.92	0.03	
					Hs_CSF2RB_3	SI00008673	CCCGTCATTCAGCTCTTCAAA	1.06	0.04	
				✓	Hs_CSF2RB_5	SI03033079	AAGCATGTCTGTGATCCACCA	1.01	0.03	
				✓	Hs_CSF2RB_6	SI03034381	AAGGACAGCCCTGTGGCTATA	0.63	0.06	
18	1441	CSF3R	0.82		Hs_CSF3R_1	SI00015001	CTGGAGCTGAGAACTACCGAA	0.99	0.02	
					Hs_CSF3R_3	SI00015015	CTCCTGCATCATCAAGCAGAA	0.98	0.03	
				✓	Hs_CSF3R_4	SI00015022	TCCTATAACTTCAGTATTGTA	0.98	0.02	
				✓	Hs_CSF3R_5	SI00156723	CCAGGCGATCTGCATACTTTA	0.68	0.04	
19	1457	CSNK2A1	0.84		Hs_CSNK2A1_8	SI02627247	CTCCTCAGTCTTGTAAATGTA	0.52	0.02	✓
				✓	Hs_CSNK2A1_9	SI02660497	TCCATTGAAGCTGAAATGGTA	0.53	0.00	✓
				✓	Hs_CSNK2A1_10	SI02660504	CTGGTCGCTTACATCACTTTA	0.81	0.02	
					Hs_CSNK2A1_11	SI03093839	CTGATCTAACCCTAAATCCAA	1.12	0.02	

Supplementary Table S1 continued

20	1495	CTNNA1	0.84		Hs_CTNNA1_6	SI02654673	AAGTGGATAAGCTGAACATTA	1.05	0.03	
					Hs_CTNNA1_7	SI02654680	CTGAGGAATGCTGGCAATGAA	1.03	0.02	
				✓	Hs_CTNNA1_8	SI02663962	GCGAATTGTGGCAGAGTGTAA	0.98	0.03	
				✓	Hs_CTNNA1_9	SI02757377	CACCCTGATGTCGCAGCCTAT	0.87	0.07	
21	1499	CTNNB1	0.84		Hs_CTNNB1_3	SI00029743	ATGGGTAGGGTAAATCAGTAA	1.18	0.04	
				✓	Hs_CTNNB1_4	SI00029750	TAAGAATTGAGTAATGGTGTA	0.79	0.03	
				✓	Hs_CTNNB1_5	SI02662478	CTCGGGATGTTCACAACCGAA	0.92	0.03	
					Hs_CTNNB1_9	SI04379662	CAGGATGATCCTAGCTATCGT	0.97	0.02	
22	6387	CXCL12	0.82		Hs_CXCL12_8	SI03043222	AGCCCGGCTGAAGAACAACAA	0.88	0.05	
					Hs_CXCL12_9	SI03049998	ATGCCGATTCTTCGAAAGCCA	0.93	0.02	
				✓	Hs_CXCL12_10	SI03649100	CTGAAGAACAACAACAGACAA	0.80	0.04	✓
				✓	Hs_CXCL12_11	SI03649149	GTGCATTGACCCGAAGCTAAA	0.82	0.03	✓
23	1605	DAG1	0.80		Hs_DAG1_1	SI00063322	AAGAACCATATTGACAGGGTA	0.66	0.02	✓
					Hs_DAG1_5	SI02633162	CCGGTGGTGAATAACAGACTA	0.84	0.01	
				✓	Hs_DAG1_6	SI02633169	AAGGGTGTGCATTACATTTCA	0.81	0.04	
				✓	Hs_DAG1_7	SI02633176	CAAGAAGATTGCCTTGGTAAA	0.77	0.01	✓
24	51473	DCDC2	0.71		Hs_DCDC2_5	SI04193644	ATGTGACACTATGGTCGAATA	1.03	0.03	
					Hs_DCDC2_6	SI04258429	CAGGTTGAGGTTCCAGTCGAT	1.09	0.02	
					Hs_DCDC2_7	SI04345901	AAGAGAGTATATGGATCGCAA	1.08	0.05	
					Hs_DCDC2_8	SI04363695	CAGGCACGCCCTGCTCGTGTA	0.97	0.02	
25	9231	DLG5	0.73		Hs_DLG5_1	SI00067858	ACGGAACTTGATACAGCACAA	0.96	0.01	
					Hs_DLG5_2	SI00067865	TTCGAGTAACTTGCAGTTCAA	0.96	0.01	
				✓	Hs_DLG5_3	SI00067872	TTGCCTGTTTGTCGACTATAA	0.99	0.06	
				✓	Hs_DLG5_4	SI00067879	CTGTGGCATATTTGTCACTAA	0.81	0.06	
26	28514	DLL1	0.83		Hs_DLL1_1	SI00369705	CACGCAGATCAAGAACACCAA	0.98	0.03	
					Hs_DLL1_2	SI00369712	CTCGGGCTGTTCAACTTCAAA	0.90	0.04	
				✓	Hs_DLL1_3	SI00369719	CCGCGTGTGCCTCAAGCACTA	1.02	0.09	
				✓	Hs_DLL1_5	SI03132836	AAGGATGAGTGCGTCATAGCA	0.54	0.03	
27	1846	DUSP4	0.85		Hs_DUSP4_3	SI00374927	TACAGTGGATTTAGAATATAT	1.05	0.06	
					Hs_DUSP4_8	SI04154850	CTGGTTCATGGAAGCCATAGA	1.07	0.04	
					Hs_DUSP4_9	SI04165056	CACTCCAACTTAGAGCAATAA	1.05	0.04	
					Hs_DUSP4_10	SI04313134	ACCGTAGCATGCAGATGTCAA	0.98	0.03	
28	8655	DYNLL1	0.78	✓	Hs_DYNLL1_3	SI03167052	CAGAATAGCCTACATTTGTAT	0.67	0.04	✓
				✓	Hs_DYNLL1_4	SI04238780	AAATCTGATGCAGATGTGTAA	0.97	0.04	
					Hs_DYNLL1_5	SI04271344	TTCGGTAGTTATGTGACACAT	1.02	0.03	
					Hs_DYNLL1_6	SI04323921	TACTAGTTTGTCGTGGTTATA	0.73	0.01	✓
29	1950	EGF	0.78		Hs_EGF_2	SI00030667	CTGGACTGATACAGGGATTAA	1.04	0.05	
					Hs_EGF_3	SI00030674	AACGATTGACTTCTTAACTGA	0.73	0.02	✓
				✓	Hs_EGF_4	SI00030681	TACGACTAATCACCTACTCAA	0.64	0.03	✓
				✓	Hs_EGF_5	SI03105613	GCGGTTGTTCCTCACCCGATA	1.00	0.04	
30	8662	EIF3B	0.79		Hs_EIF3S9_1	SI00377825	CGGGAAGATTGAACTCATCAA	1.01	0.04	
					Hs_EIF3S9_5	SI04196136	CACTGACGAGCTGGACAGCAA	0.86	0.04	
					Hs_EIF3S9_6	SI04285722	CACGGTCATGAACATCGCAGA	0.85	0.03	
					Hs_EIF3S9_7	SI04337613	GACCGACTTGAGAAACTCAAA	1.02	0.00	
31	2036	EPB41L1	0.62		Hs_EPB41L1_1	SI00097566	ACGGATGTGCTTTGAAGTTAT	1.02	0.01	
					Hs_EPB41L1_2	SI00097573	CCCGCCAAATAAGAAACGCAA	1.00	0.00	
				✓	Hs_EPB41L1_5	SI00160489	CAGAGTCTCCGCTATGGATAA	0.79	0.05	✓
				✓	Hs_EPB41L1_7	SI02639014	CACGTTACAGTTAGCAGACGA	0.82	0.04	✓
32	2037	EPB41L2	0.81	✓	Hs_EPB41L2_3	SI00380247	CAGGCTAAGGGTGATGCTGAA	0.71	0.03	✓
				✓	Hs_EPB41L2_4	SI00380254	TCCCATGCATTTAATATATTA	0.90	0.01	
					Hs_EPB41L2_7	SI04294962	CACGAAGGCGCTCTTAAGCAA	0.94	0.05	
					Hs_EPB41L2_8	SI04322409	TGCAGCCTACTGAATTAGTAA	0.84	0.02	✓
33	1969	EPHA2	0.73		Hs_EPHA2_5	SI00300181	AAGGAAGTGGTACTGCTGGAC	1.07	0.02	
					Hs_EPHA2_6	SI00300188	AAGCGCCTGTTCACCAAGATT	0.99	0.04	
				✓	Hs_EPHA2_7	SI02223508	CAGCGCCAAGTAAACAGGGTA	0.83	0.01	
				✓	Hs_EPHA2_8	SI02223515	TCGGACAGACATATAGGATAT	0.96	0.03	
34	2044	EPHA5	0.77		Hs_EPHA5_4	SI00063686	ACCAGCTACACGATTATCAAA	1.11	0.01	
				✓	Hs_EPHA5_6	SI00287511	CCCGGCAGTATGTGTCTGTAA	0.65	0.02	✓
				✓	Hs_EPHA5_5	SI02223536	ACCAGTTGGATCTCCAATGAA	0.59	0.03	✓
					Hs_EPHA5_8	SI03649380	TTGGCAGAACATAGCCCACTA	0.83	0.05	
35	2066	ERBB4	0.80		Hs_ERBB4_4	SI00074214	CTGGAGAATTTACGCATTATT	1.26	0.07	
				✓	Hs_ERBB4_5	SI02223893	CTACGTGTTAGTGGCTCTTAA	0.67	0.02	
				✓	Hs_ERBB4_6	SI02223900	TCGGGATTTGGCAGCCCGTAA	0.87	0.03	
					Hs_ERBB4_10	SI04435067	CAAGCATTGGATAATCCCGAA	1.01	0.02	
36	60412	EXOC4	0.84	✓	Hs_EXOC4_1	SI03227595	TAGCCGAGTTGTGCAGCGTAA	1.06	0.02	
					Hs_EXOC4_2	SI04242203	CACCGGCACCTGTACATCAAA	1.06	0.03	
					Hs_EXOC4_3	SI04289411	AACGTTACTGTGGAGAACCAA	1.09	0.02	
					Hs_EXOC4_4	SI04316354	TAAGGGCTTGGCGAAACTGAA	0.88	0.07	
37	2263	FGFR2	0.81		Hs_FGFR2_3	SI00002940	CGGAGGAGCGTTGCCATTCAA	1.01	0.06	
				✓	Hs_FGFR2_6	SI02623047	TTAGTTGAGGATACCACATTA	0.83	0.02	
				✓	Hs_FGFR2_7	SI02665299	CAGCATATGTGTAAAGATTTA	0.91	0.02	
					Hs_FGFR2_12	SI04380649	CCCATCTGACAAGGGAAATTA	1.03	0.04	
38	2549	GAB1	0.59		Hs_GAB1_1	SI00031899	CACGAGCATTTCCAAGTGATA	0.97	0.02	
					Hs_GAB1_3	SI00031913	CAGGACCTAGTTCTCTGGTTA	0.75	0.11	✓
				✓	Hs_GAB1_6	SI02654736	TAGATGCTGGATTGACATTTA	0.77	0.02	
				✓	Hs_GAB1_7	SI03077403	CCCGACCAGATTCAGTGCATA	0.64	0.12	✓
39	2597	GAPDH	0.85	✓	Hs_GAPD_5	SI02653266	CCGAGCCACATCGCTCAGACA	0.91	0.07	
					Hs_GAPD_7	SI02655926	CTCCTCTGACTTCAACAGCGA	1.10	0.06	
				✓	Hs_GAPDH_3	SI03571113	AAGGTCGGAGTCAACGGATTT	0.94	0.05	
					Hs_GAPDH_6	SI04220279	CAGGAGCGAGATCCCTCCAAA	1.13	0.02	
40	2690	GHR	0.82		Hs_GHR_3	SI00003444	TAGAACCTAATTCAATTCAAA	1.05	0.01	
					Hs_GHR_4	SI00003451	TCCCAAGAGCTACGTATTTAA	0.93	0.01	
				✓	Hs_GHR_5	SI03037090	AAGTGTTAATCCAGGCCTAAA	0.84	0.01	
				✓	Hs_GHR_6	SI03072104	CAGGTGAGCGACATTACACCA	0.91	0.03	

Supplementary Table S1 continued

41	2775	GNAO1	0.76		Hs_GNAO1_2	SI00128632	CAGCGCCGCCAAAGACGTGAA	0.90	0.02	
					Hs_GNAO1_4	SI00128646	CAGGTGCTCCACGAAGACGAA	0.76	0.06	
				✓	Hs_GNAO1_7	SI03050523	ATGGACACTTTGGGCATCGAA	0.97	0.04	
					Hs_GNAO1_9	SI04658416	CGGCTCCAGCCTCGACTATTA	1.04	0.04	
42	10750	GRAP	0.84		Hs_GRAP_1	SI00430668	CAACATCAGGATGTCCGTCAA	0.69	0.04	✓
					Hs_GRAP_5	SI03156279	CAACGAGCTGGTCGACTTCTA	1.06	0.03	
				✓	Hs_GRAP_7	SI03189088	CCGCCGTGGCGACATCATTGA	0.82	0.04	✓
					Hs_GRAP_9	SI04180519	CAGGAAGGTTGAGGACTCCCA	1.00	0.05	
43	2885	GRB2	0.66		Hs_GRB2_1	SI00032550	CTGGTATTCTCTCTATGCAAA	0.93	0.05	
					Hs_GRB2_3	SI00032564	TAGGTAGTGATTAACTGTGAA	0.91	0.10	
				✓	Hs_GRB2_5	SI00300328	AAGTTTGGAAACGATGTGCAG	0.65	0.02	✓
				✓	Hs_GRB2_8	SI02654750	CAAGAACTACATAGAAATGAA	0.67	0.08	✓
44	3280	HES1	0.84		Hs_HES1_2	SI00078330	CTGGTGCTGATAACAGCGGAA	1.17	0.05	
					Hs_HES1_3	SI00078337	CACGACACCGGATAAACCAAA	1.00	0.01	
				✓	Hs_HES1_4	SI00078344	AAAGACGAAGAGCAAGAATAA	1.00	0.02	
				✓	Hs_HES1_5	SI03075016	CCAGATCAATGCCATGACCTA	0.64	0.04	
45	3320	HSP90AA1	0.77		Hs_HSPCA_4	SI00075971	ATGGCATGACAACTACTTTAA	0.88	0.07	
					Hs_HSPCA_5	SI02635024	CAGAATGAAGGAGAACCAGAA	1.09	0.01	
				✓	Hs_HSP90AA1_1	SI03028606	AACCCTGACCATTCCATTATT	0.86	0.05	
				✓	Hs_HSP90AA1_2	SI03117814	TGCACTGTAAGACGTATGTAA	0.71	0.02	
46	7184	HSP90B1	0.72		Hs_TRA1_5	SI00302008	AAGTTGATGTGGATGGTACAG	0.76	0.03	
					Hs_TRA1_6	SI02630838	ATGGATTAAATGCATCACAAA	1.02	0.02	
				✓	Hs_TRA1_8	SI02655177	AAGTTGATGTGGATGGTACAT	0.89	0.03	
				✓	Hs_TRA1_9	SI02663738	TCGCCTCAGTTTGAACATTGA	0.96	0.03	
47	3717	JAK2	0.82		Hs_JAK2_4	SI00070742	ATCACTAGATACATATCTGAA	0.94	0.04	
					Hs_JAK2_6	SI02634177	CAGAATTAGCAAACCTTATAA	0.73	0.06	✓
				✓	Hs_JAK2_7	SI02659657	AGCCATCATACGAGATCTTAA	0.86	0.02	
				✓	Hs_JAK2_8	SI02659664	CTGCCTTACGATGACAGAAAT	0.80	0.03	✓
48	3728	JUP	0.72		Hs_JUP_1	SI00034720	ATGCGTAACTACAGTTATGAA	0.80	0.09	✓
					Hs_JUP_2	SI00034727	CCAGAGCATGATTCCCATCAA	0.74	0.03	✓
				✓	Hs_JUP_3	SI00034734	AACCATCGGCTTGATCAGGAA	1.05	0.02	
				✓	Hs_JUP_4	SI00034741	CCGCATCTCCGAGGACAAGAA	0.93	0.05	
49	3791	KDR	0.78		Hs_KDR_4	SI00035259	TACCAGTACGGCACCACTCAA	0.92	0.03	
				✓	Hs_KDR_5	SI00605528	AACGCTGACATGTACGGTCTA	0.62	0.01	
				✓	Hs_KDR_6	SI00605535	AAGGCTAATACAACTCTTCAA	0.91	0.04	
					Hs_KDR_8	SI03649436	CAGGGACCTGGCGGCACGAAA	0.90	0.04	
50	7071	KLF10	0.79		Hs_KLF10_4	SI00079926	CCGGCGCCATCTATCAGCCAA	1.01	0.02	
					Hs_KLF10_5	SI02635668	ATGGCTTATGTCATCAGTAAA	1.06	0.04	
				✓	Hs_KLF10_8	SI03043236	AGCCCGTTGTGCAGAGTTCAA	0.80	0.08	✓
				✓	Hs_KLF10_9	SI03082268	CCGGCGGTTCATGAGGAGTGA	0.72	0.04	✓
51	283455	KSR2	0.82	✓	Hs_KSR2_5	SI02665544	CAGGCTTACCGTGGACGCCTA	0.83	0.02	
				✓	Hs_KSR2_6	SI02665551	AAGGAAATCCATTACTTCAAA	1.02	0.04	
					Hs_KSR2_7	SI04379158	CTGGCTCGACTGGAGCATTAA	0.93	0.03	
					Hs_KSR2_8	SI04379165	AACCCACGAGCACTTAATATA	0.99	0.01	
52	63920	LOC63920	0.70	✓	Hs_LOC63920_5	SI03121783	AAAGAGATACCTCATATCGTA	1.06	0.03	
					Hs_LOC63920_6	SI04156558	TTCTCGGACATCATTGAACAA	1.01	0.03	
					Hs_LOC63920_7	SI04270252	TGCCATGTCGAAGAAACGCAA	1.01	0.04	
					Hs_LOC63920_8	SI04370149	AAGAGCAGTAATTTAGTTGAT	1.08	0.03	
53	5599	MAPK8	0.76		Hs_MAPK8_9	SI00300783	AAGAAGCTAAGCCGACCATTT	1.01	0.02	
					Hs_MAPK8_12	SI02757209	GTGGAAAGAATTGATATATAA	1.07	0.03	
				✓	Hs_MAPK8_14	SI02758644	ATGAAATGTGTTAATCACAAA	0.95	0.01	
				✓	Hs_MAPK8_15	SI02758651	ATGATGTGTCTTCAATGTCAA	0.69	0.06	
54	4739	NEDD9	0.83		Hs_NEDD9_1	SI00657363	CACCCAAGAACAAGAGGTATA	0.90	0.02	
					Hs_NEDD9_2	SI00657370	CGCTGCCGAAATGAAGTATAA	0.67	0.02	
				✓	Hs_NEDD9_5	SI03166898	CAGAAGCTCTATCAAGTGCCA	0.62	0.05	✓
				✓	Hs_NEDD9_6	SI03192406	CCTGACCGTCATAGAGCAGAA	0.58	0.03	✓
55	25983	NGDN	0.82		Hs_C14orf120_2	SI00318584	ACGCTTGGTTCCAGTACATTA	0.92	0.03	
					Hs_NGDN_1	SI04253305	CAGCAATATGATGAGCAAGTT	0.75	0.13	
					Hs_NGDN_2	SI04316585	CTGGTGCCTATCCTACAGAAA	0.87	0.04	
				✓	Hs_NGDN_3	SI04367349	ATGAGGATCAGAATCCTATTA	1.00	0.01	
56	5063	PAK3	0.78		Hs_PAK3_4	SI00039851	CCCTCATGAGTAAGACTGAAA	0.76	0.01	✓
				✓	Hs_PAK3_5	SI00287434	TTCCAGTACTTTGTACAGGAA	0.72	0.05	✓
				✓	Hs_PAK3_6	SI02628983	CAAGAAGGAATTAATTATTAA	0.95	0.00	
					Hs_LOC100129569_4	SI04808300	CTGGGATGAGCAAGGTGTAAA	0.85	0.00	
57	50855	PARD6A	0.84		Hs_PARD6A_2	SI00117873	GACGGACATGATGGTTGCCAA	1.08	0.05	
					Hs_PARD6A_3	SI00117880	CGAGGTGAAGAGCAAATTTGA	0.88	0.01	
				✓	Hs_PARD6A_5	SI02664340	CCAGGTTTCCTCAGTCATAGA	0.81	0.02	✓
				✓	Hs_PARD6A_6	SI03048619	ATCGTCGAGGTGAAGAGCAAA	0.83	0.03	✓
58	5159	PDGFRB	0.83		Hs_PDGFRB_4	SI00040383	ATCCATCAACGTCTCTGTGAA	0.92	0.02	
				✓	Hs_PDGFRB_5	SI00605738	CCGAGCAACTTTGATCAACGA	0.75	0.02	✓
				✓	Hs_PDGFRB_6	SI00605745	CTGCCGAGCAACTTTGATCAA	0.65	0.04	✓
					Hs_PDGFRB_8	SI03034773	AAGGAGGACCCATCTATATCA	0.93	0.01	
59	5296	PIK3R2	0.73		Hs_PIK3R2_2	SI00071547	CGGCGTGAGGGCAACGAGAAA	0.83	0.08	✓
				✓	Hs_PIK3R2_5	SI00287532	CCGCGAGTATGACCAGCTTTA	1.00	0.10	
				✓	Hs_PIK3R2_6	SI00287539	TTGGTACGTGGGCAAGATCAA	0.64	0.05	✓
					Hs_PIK3R2_7	SI04378983	CTCGGACAACAGAGAGATCGA	1.00	0.04	
60	23236	PLCB1	0.84	✓	Hs_PLCB1_5	SI02780939	TCGAGATTACATGGATGTTAA	0.88	0.02	
				✓	Hs_PLCB1_6	SI02781184	CAGAGATGATCCGGTCATATA	0.74	0.04	
					Hs_PLCB1_9	SI04439106	TTCGGCCAGGCTATCACTATA	0.96	0.02	
					Hs_PLCB1_10	SI04439113	AAGGGTCAAACATCGAGATTA	1.04	0.04	
61	5336	PLCG2	0.81		Hs_PLCG2_4	SI00041223	GACGCGGACTACCAAGATCAA	0.85	0.02	
				✓	Hs_PLCG2_5	SI02662681	GACGACGGTTGTGAATGATAA	0.82	0.02	✓
				✓	Hs_PLCG2_7	SI03106138	GGGCGGGACCCTGAAATACTA	0.81	0.01	
					Hs_PLCG2_10	SI04380950	CAGAGGTTTCTCATACATGAA	0.69	0.03	✓

Supplementary Table S1 continued

62	5337	PLD1	0.80	✓	Hs_PLD1_3	SI00686357	ATGGGATATTTGATTACGTAA	0.76	0.04	
					Hs_PLD1_6	SI04166134	CAGGCCTGACGCATTCTCGTA	0.98	0.01	
					Hs_PLD1_7	SI04271176	TACCGGGTATATGTCGTGATA	1.00	0.02	
					Hs_PLD1_8	SI04369316	ACCGGCGGAGGAAATGCTCTA	0.98	0.05	
63	5359	PLSCR1	0.77		Hs_PLSCR1_3	SI00129332	ACGTAAGATTCAGGAAACGAA	1.08	0.13	
					Hs_PLSCR1_4	SI00129339	CGGGTGTTGGCGCAAAGGTTA	1.05	0.09	
				✓	Hs_PLSCR1_5	SI03067043	CAGCGCCACAGCCTCCATTAA	0.66	0.04	✓
				✓	Hs_PLSCR1_6	SI03075751	CCAGTGTATAATCAGCCAGTA	0.82	0.06	✓
64	5456	POU3F4	0.73		Hs_POU3F4_4	SI00006748	ACAGTTCGCCAAACAATTCAA	0.95	0.01	
					Hs_POU3F4_5	SI03021214	TGGTTCTGTAATCGAAGACAA	0.98	0.04	
				✓	Hs_POU3F4_6	SI03062675	CAGAAACTTCTCCAAAGTGAT	0.89	0.05	
				✓	Hs_POU3F4_7	SI03077410	CCCGACCAGCATTGACAAGAT	0.94	0.02	
65	5578	PRKCA	0.81		Hs_PRKCA_5	SI00301308	AACCATCCGCTCCACACTAAA	0.82	0.02	✓
				✓	Hs_PRKCA_6	SI00605927	CGCAGTGGAATGAGTCCTTTA	0.76	0.04	✓
				✓	Hs_PRKCA_7	SI00605934	TACAAGTTGCTTAACCAAGAA	1.05	0.03	
					Hs_PRKCA_8	SI02629368	CAGCTGGTTATTGCTAACATA	0.89	0.01	
66	5581	PRKCE	0.81	✓	Hs_PRKCE_5	SI00287784	CCCGACCATGGTAGTGTTCAA	0.76	0.05	✓
				✓	Hs_PRKCE_6	SI02622088	CACGGAAACACCCGTACCTTA	0.73	0.01	✓
					Hs_PRKCE_7	SI03059658	CACGACGAGTTCGTCACCGAT	1.00	0.03	
					Hs_PRKCE_10	SI04379424	AAGAGTGTATGTGATCATCGA	0.95	0.03	
67	2889	RAPGEF1	0.62		Hs_RAPGEF1_2	SI00075621	CCGGGTTTAGGACGTGGTCAA	0.91	0.05	
					Hs_RAPGEF1_3	SI00075628	GCAGGTCTTGTTGCCAATTTA	0.88	0.01	
				✓	Hs_RAPGEF1_5	SI02634989	CAGGAAAGATTTGGTGTTGTA	0.64	0.04	✓
				✓	Hs_RAPGEF1_7	SI03099194	CTGGGTCCGGTCCATAATCAT	0.78	0.01	✓
68	9252	RPS6KA5	0.80	✓	Hs_RPS6KA5_8	SI02225433	AAGCCAGTCATTCGAGATGAA	0.68	0.02	✓
				✓	Hs_RPS6KA5_9	SI02225440	CTGGATCTCTTACGTAATTCA	0.73	0.03	
					Hs_RPS6KA5_11	SI03025232	TTGGGATGATTTAGCCGCCAA	1.01	0.25	
					Hs_RPS6KA5_12	SI03025428	TTGGGTGTTCTAATGTATGAA	0.68	0.03	✓
69	10045	SH2D3A	0.85		Hs_SH2D3A_3	SI00078036	CGGCTCTGGTTCACAGTTATA	1.06	0.03	
					Hs_SH2D3A_5	SI03071236	CAGGGCAAGGAAGTGGAGCAA	0.94	0.05	
				✓	Hs_SH2D3A_6	SI03072167	CAGGTGCCACAGGATGGAGAA	0.88	0.05	
				✓	Hs_SH2D3A_7	SI03105074	GCCGTGATTTCTAGCAGTTGA	1.11	0.02	
70	25759	SHC2	0.72		Hs_SHC2_3	SI00717801	AAGGATCTGTTTGACATGCGA	1.01	0.01	
				✓	Hs_SHC2_6	SI02825256	ACGGAACCATTTCGCCTTTAA	0.77	0.06	
				✓	Hs_SHC2_7	SI02825263	TTCGGATCGGTTTGTAATTAA	0.95	0.03	
					Hs_SHC2_12	SI04280220	AGTGAGGGTGTTCATACCAAA	1.00	0.02	
71	80725	SNIP	0.75	✓	Hs_SNIP_3	SI00728161	CAGGCCACTAAACCATCTAAA	0.76	0.07	✓
				✓	Hs_SNIP_5	SI03233727	TCGCATCATCGCAGAGCTAGA	0.94	0.03	
					Hs_SNIP_6	SI04132849	CCCGCTGTACGGCGACGGCTA	0.83	0.06	✓
					Hs_SNIP_7	SI04191481	AAGGACCTCATAAATGATGCA	1.09	0.02	
72	8651	SOCS1	0.77		Hs_SOCS1_1	SI00055370	CTGGTTGTTGTAGCAGCTTAA	0.88	0.04	
					Hs_SOCS1_4	SI00055391	TAGGATGGTAGCACACAACCA	1.03	0.03	
				✓	Hs_SOCS1_5	SI03107342	TAAAGTCAGTTTAGGTAATAA	0.99	0.04	
				✓	Hs_SOCS1_6	SI03108812	TACCCAGTATCTTTGCACAAA	0.74	0.01	
73	6655	SOS2	0.81		Hs_SOS2_5	SI04166631	CCCACTCTCTCAGCTAATGAA	0.68	0.02	
					Hs_SOS2_6	SI04189416	TTGGACGAAGCTGTGGAATTA	1.07	0.01	
					Hs_SOS2_7	SI04307436	CTTCATTATCGTAGTACTCTA	0.87	0.03	
					Hs_SOS2_8	SI04349884	CTGAGATTACCAAGTCCTGAA	1.06	0.03	
74	92521	SPECC1	0.69		Hs_HCMOGT-1_3	SI00434770	CAGGCTTCGAAGTGAACTAAA	0.98	0.01	
					Hs_SPECC1_2	SI04160646	CAGCACCGGGCTGTCAAGTTA	0.93	0.02	
					Hs_SPECC1_3	SI04201141	TCAAGTAGCGATGTTACCAAA	1.04	0.00	
					Hs_SPECC1_4	SI04266983	CAAGAATTATCAGACCAGCAA	0.92	0.03	
75	6714	SRC	0.79		Hs_SRC_6	SI02223921	AAGCAGTGCCTGCCTATGAAA	0.70	0.03	✓
				✓	Hs_SRC_7	SI02223928	CGGCTTGTGGGTGATGTTTGA	0.65	0.02	✓
				✓	Hs_SRC_10	SI02664151	CTCCATGTGCGTCCATATTTA	1.03	0.02	
					Hs_SRC_11	SI03041605	ACGGCGCGGCAAGGTGCCAAA	1.00	0.01	
76	79718	TBL1XR1	0.75	✓	Hs_TBL1XR1_1	SI00136808	AAGGAGCTATCTGTTTATGTA	0.97	0.04	
					Hs_TBL1XR1_9	SI02645727	TAGATACTAGATTGTATTGAA	1.04	0.01	
					Hs_TBL1XR1_10	SI03025925	TTGTTTGATGGTCGACCAATA	0.86	0.09	
				✓	Hs_TBL1XR1_11	SI03110905	TAGCACCTTAGGGCAGCATAA	0.80	0.06	
77	7205	TRIP6	0.75		Hs_TRIP6_1	SI00050421	CACGGGCTCCCTGAAGCCAAA	1.04	0.00	
					Hs_TRIP6_2	SI00050428	CTGTATGACTTTGTCACCAAA	1.02	0.06	
				✓	Hs_TRIP6_4	SI02630866	TGGGCTGCTTTGTATGTTCTA	0.99	0.04	
				✓	Hs_TRIP6_5	SI03069654	CAGGAGGAGACTGTGAGAATT	0.78	0.08	
78	54822	TRPM7	0.70		Hs_TRPM7_5	SI03048465	ATCGGAGGTCTGGCCGAAATA	0.99	0.03	
					Hs_TRPM7_6	SI03079468	CCCTGACGGTAGATACATTAA	0.78	0.11	✓
				✓	Hs_TRPM7_7	SI03083549	CCTGTAAGATCTATCGTTCAA	0.96	0.02	
				✓	Hs_TRPM7_8	SI03095806	CTGCTAGCGTATATTCATAAA	0.65	0.05	✓
79	7409	VAV1	0.83		Hs_VAV1_2	SI00077007	CAGCATTAAATATAACGTCGA	0.99	0.02	
					Hs_VAV1_3	SI00077014	CAGGTGGAGTCAGCCAGCAAA	0.94	0.04	
				✓	Hs_VAV1_4	SI00077021	GTCGAGGTCAAGCACATTAAA	0.82	0.03	✓
				✓	Hs_VAV1_5	SI03054401	CAAGGAGAGGTTCCTCGTCTA	0.72	0.04	✓
80	9948	WDR1	0.84	✓	Hs_WDR1_5	SI03122448	AAAGTGCGTCATCCTAAGGAA	0.93	0.02	
					Hs_WDR1_6	SI04186756	CTCCCTGTCCGGGTACATCAA	1.02	0.02	
					Hs_WDR1_7	SI04195254	CAGCGGACAAGGAGTTGTGAA	0.95	0.05	
					Hs_WDR1_8	SI04247922	CAGCCTCATGCTGCGGGACTA	0.94	0.02	
81	7520	XRCC5	0.78		Hs_XRCC5_4	SI00129675	ACCCTCATAAGTCGTCACTAA	0.78	0.07	✓
				✓	Hs_XRCC5_6	SI02663766	AAGCATAACTATGAGTGTTTA	0.92	0.04	
				✓	Hs_XRCC5_7	SI02663773	AAGCGAGTAACCAGCTCATAA	0.85	0.04	✓
					Hs_XRCC5_8	SI03022271	TTCCGCTATGGAAGTGATATA	1.05	0.04	
82	7525	YES1	0.81		Hs_YES1_5	SI00302218	AATCCCTCCATGAATTGATGA	0.96	0.02	
				✓	Hs_YES1_6	SI02223935	CCAGCCTACATTCACTTCTAA	0.75	0.02	✓
				✓	Hs_YES1_7	SI02223942	GAGGCTCCTGCTTATTTATAA	0.81	0.03	✓
					Hs_YES1_8	SI02635206	AAGTATAATGCAGTACATTAA	0.91	0.01	

Supplementary Table S1 continued

83	8882	ZNF259	0.71		Hs_ZNF259_9	SI04197018	TGAGATGAAGGTGGAGCGTTA	1.03	0.03	
					Hs_ZNF259_10	SI04203570	TTGAATTGGCCTAGGAGGAAA	0.76	0.04	
					Hs_ZNF259_11	SI04344487	GTCGCTATGCATGAACTGTTA	1.06	0.01	
					Hs_ZNF259_12	SI04349156	CTGAAGGAGCTAAAGCAAGTA	0.89	0.03	
84	7791	ZYX	0.84	✓	Hs_ZYX_1	SI00302225	AAGGTGAGCAGTATTGATTTG	0.75	0.02	
					Hs_ZYX_3	SI02651320	CAGAATGTGGCTGTCAACGAA	1.08	0.04	
					Hs_ZYX_6	SI02651341	CAGTATTGATTTGGAGATCGA	1.00	0.05	
					Hs_ZYX_8	SI04209856	CCAGGTGGATCTGGGTCACAA	0.95	0.04	
