# Supplementary material for: A Synthetic Lethality Screen Using a Focused siRNA Library to Identify Sensitizers to Dasatinib Therapy for the Treatment of Epithelial Ovarian Cancer
Source: PLoS One. 2015 Dec 4;10(12):e0144126. doi: 10.1371/journal.pone.0144126 (PMC4670180; doi:10.1371/journal.pone.0144126)
Supplement: S2 Table — The 31 genes selected for additional studies are indicated. (PDF) [file pone.0144126.s005.pdf]

S2 Table

	Gene ID	Gene Symbol	Percent Reduction in Gene Expression	Average Ct Value from Negative Control Wells	Selected for Further Studies
1	25	ABL1	88%	24.9	✓
2	309	ANXA6	92%	23.4	✓
3	8412	BCAR3	81%	25.9	✓
4	602	BCL3	85%	27.9	✓
5	805	CALM2	64%	27.9	
6	896	CCND3	97%	26.7	✓
7	919	CD247	Undetermined	>35 cycles	
8	916	CD3E	Undetermined	>35 cycles	
9	1398	CRK	87%	26.4	✓
10	1457	CSNK2A1	87%	24.0	✓
11	6387	CXCL12	Undetermined	>35 cycles	
12	1605	DAG1	91%	24.7	✓
13	8655	DYNLL1	92%	25.9	✓
14	1950	EGF	74%	29.8	✓
15	2036	EPB41L1	94%	25.7	✓
16	2037	EPB41L2	76%	24.9	✓
17	2044	EPHA5	Increased expression	31.5	
18	2549	GAB1	75%	26.7	✓
19	10750	GRAP	Undetermined	>35 cycles	
20	2885	GRB2	92%	24.0	✓
21	3717	JAK2	78%	28.9	✓
22	3728	JUP	86%	25.4	✓
23	7071	KLF10	82%	25.9	✓
24	4739	NEDD9	78%	28.1	✓
25	5063	PAK3	86%	31.0	✓
26	50855	PARD6A	97%	31.0	✓
27	5159	PDGFRB	79%	27.7	✓
28	5296	PIK3R2	Increased expression	27.6	
29	5336	PLCG2	73%	29.4	✓
30	5359	PLSCR1	90%	26.0	✓
31	5578	PRKCA	84%	25.0	✓
32	5581	PRKCE	79%	27.9	✓
33	2889	RAPGEF1	94%	26.0	✓
34	9252	RPS6KA5	62%	29.6	
35	80725	SNIP	58%	26.0	
36	6714	SRC	85%	25.3	✓
37	54822	TRPM7	81%	27.9	✓
38	7409	VAV1	74%	27.9	✓
39	7520	XRCC5	74%	22.8	✓
40	7525	YES1	95%	26.0	✓
