# Supplementary material for: A Synthetic Lethality Screen Using a Focused siRNA Library to Identify Sensitizers to Dasatinib Therapy for the Treatment of Epithelial Ovarian Cancer
Source: PLoS One. 2015 Dec 4;10(12):e0144126. doi: 10.1371/journal.pone.0144126 (PMC4670180; doi:10.1371/journal.pone.0144126)
Supplement: S3 Table — The two genes highlighted in yellow appear to synergize with both drugs (SI ≤ 0.85) and therefore we did not use them for further studies. The remaining 29 hits were considered as dasatinib-specific sensitizers. Correlation of hits–The Spearman coefficient (r) and statistical significance (two-tailed t-test) for 29 genes were calculated using GraphPad Prism to determine the correlation of gene expression with dasatinib sensitivity. Genes highlighted in green show statistically significant correlation of basal gene expression with dasatinib sensitivity. ND, not determined. (PDF) [file pone.0144126.s006.pdf]

S3 Table

Specificity of Hits					Correlation of Hits	
	Gene ID	Gene Symbol	Dasatinib Avg SI Value ± SEM (n = 2)	Imatinib Avg SI Value ± SEM (n = 2)	Spearman Correlation Coefficient, r	P Value
1	2549	GAB1	0.31 ± 0.01	0.84 ± 0.13	ND	ND
2	2036	EPB41L1	0.40 ± 0.03	0.94 ± 0.10	0.39	0.3956
3	8412	BCAR3	0.44 ± 0.03	0.91 ± 0.04	0.86	0.0238
4	1457	CSNK2A1	0.46 ± 0.02	0.88 ± 0.05	-0.82	0.0341
5	1605	DAG1	0.48 ± 0.03	0.95 ± 0.03	-0.54	0.2357
6	896	CCND3	0.49 ± 0.01	0.92 ± 0.05	-0.46	0.3024
7	2889	RAPGEF1	0.49 ± 0.02	0.91 ± 0.13	-0.68	0.1095
8	3728	JUP	0.50 ± 0.02	0.82 ± 0.10	ND	ND
9	1950	EGF	0.51 ± 0.01	0.91 ± 0.03	0.04	0.9635
10	5159	PDGFRB	0.53 ± 0.03	0.96 ± 0.04	-0.29	0.556
11	4739	NEDD9	0.53 ± 0.06	1.07 ± 0.13	0.68	0.1095
12	6714	SRC	0.54 ± 0.03	0.98 ± 0.16	-0.14	0.7825
13	25	ABL1	0.54 ± 0.04	0.97 ± 0.13	-0.46	0.3024
14	602	BCL3	0.56 ± 0.04	1.00 ± 0.01	0.43	0.3536
15	1398	CRK	0.57 ± 0.06	1.08 ± 0.10	0.46	0.3024
16	5359	PLSCR1	0.59 ± 0.03	0.87 ± 0.08	-0.04	0.9635
17	7409	VAV1	0.59 ± 0.04	0.99 ± 0.01	0.64	0.1389
18	5063	PAK3	0.61 ± 0.04	0.96 ± 0.06	0.43	0.4194
19	3717	JAK2	0.63 ± 0.01	0.92 ± 0.08	-0.54	0.2357
20	5336	PLCG2	0.63 ± 0.03	1.01 ± 0.10	0.18	0.7131
21	8655	DYNLL1	0.64 ± 0.03	0.92 ± 0.03	-0.09	0.8397
22	2037	EPB41L2	0.64 ± 0.04	0.94 ± 0.14	-0.07	0.9063
23	54822	TRPM7	0.66 ± 0.03	0.97 ± 0.06	-0.04	0.9635
24	7071	KLF10	0.68 ± 0.02	1.01 ± 0.06	-0.54	0.2357
25	50855	PARD6A	0.68 ± 0.02	0.98 ± 0.01	-0.39	0.3956
26	309	ANXA6	0.68 ± 0.06	0.99 ± 0.14	-0.39	0.3956
27	5581	PRKCE	0.69 ± 0.03	0.97 ± 0.09	-0.96	0.0028
28	2885	GRB2	0.69 ± 0.05	0.97 ± 0.15	-0.71	0.0881
29	7525	YES1	0.70 ± 0.01	0.91 ± 0.01	-0.54	0.2357
30	5578	PRKCA	0.76 ± 0.04	1.00 ± 0.08	0.93	0.0067
31	7520	XRCC5	0.77 ± 0.01	0.90 ± 0.03	-0.14	0.7825
