# Supplementary material for: A Synthetic Lethality Screen Using a Focused siRNA Library to Identify Sensitizers to Dasatinib Therapy for the Treatment of Epithelial Ovarian Cancer
Source: PLoS One. 2015 Dec 4;10(12):e0144126. doi: 10.1371/journal.pone.0144126 (PMC4670180; doi:10.1371/journal.pone.0144126)
Supplement: S4 Table — Shown are the hits ranked by the average fold-change in gene expression in the tumor samples relative to the control tissue. Data from multiple probes are shown when available. (PDF) [file pone.0144126.s007.pdf]

S4 Table

Gene ID	Gene Symbol	Agilent Probe ID	Probe	Average Expression in Tumor Tissue from Patients with Serous Ovarian Carcinoma	SEM	Average Expression in Organ Specific Healthy Control Tissue	SEM	Fold Change (Tumor: Healthy)	T-test, p value
1605	DAG1	A_24_P225961	Probe 1	0.821	0.02	0.374	0.04	2.20	1.9E-06
7409	VAV1	A_23_P38959		0.658	0.02	0.359	0.04	1.83	6.6E-05
1457	CSNK2A1	A_24_P76666	Probe 1	0.554	0.01	0.310	0.04	1.78	2.1E-04
1457	CSNK2A1	A_23_P502575	Probe 2	1.457	0.03	0.824	0.05	1.77	1.2E-07
7525	YES1	A_23_P164507	Probe 2	0.688	0.02	0.428	0.05	1.61	5.2E-04
2885	GRB2	A_24_P39654	Probe 2	0.665	0.01	0.417	0.04	1.59	1.8E-04
54822	TRPM7	A_23_P88470	Probe 1	0.708	0.02	0.482	0.05	1.47	3.8E-03
54822	TRPM7	A_24_P50829	Probe 2	1.291	0.04	0.969	0.11	1.33	2.2E-02
1605	DAG1	A_23_P212579	Probe 2	1.512	0.02	1.177	0.09	1.28	5.6E-03
2036	EPB41L1	A_24_P185709	Probe 1	2.321	0.06	1.843	0.29	1.26	1.4E-01
7520	XRCC5	A_24_P345498		0.800	0.01	0.646	0.03	1.24	5.7E-04
5578	PRKCA	A_23_P55099	Probe 1	0.580	0.02	0.475	0.05	1.22	1.0E-01
5581	PRKCE	A_23_P250564	Probe 2	2.065	0.04	1.751	0.05	1.18	2.7E-04
25	ABL1	A_24_P282416	Probe 5	0.425	0.01	0.364	0.03	1.17	1.1E-01
602	BCL3	A_23_P4662		2.767	0.08	2.376	0.54	1.16	5.0E-01
5581	PRKCE	A_23_P348194	Probe 1	1.319	0.02	1.143	0.09	1.15	1.0E-01
7525	YES1	A_24_P48403	Probe 1	0.981	0.05	0.853	0.06	1.15	1.0E-01
2885	GRB2	A_23_P77847	Probe 1	0.799	0.01	0.707	0.02	1.13	2.9E-03
1398	CRK	A_23_P83556	Probe 2	0.838	0.02	0.745	0.08	1.13	3.0E-01
2885	GRB2	A_24_P407717	Probe 3	0.556	0.01	0.510	0.05	1.09	3.9E-01
6714	SRC	A_23_P308603		1.678	0.03	1.613	0.08	1.04	4.7E-01
3717	JAK2	A_23_P123608		0.726	0.02	0.704	0.06	1.03	7.4E-01
1398	CRK	A_24_P270814	Probe 1	0.843	0.02	0.822	0.08	1.03	7.9E-01
896	CCND3	A_23_P361773		0.736	0.02	0.719	0.11	1.02	8.8E-01
5336	PLCG2	A_23_P106675		2.198	0.06	2.188	0.34	1.00	9.8E-01
8412	BCAR3	A_23_P97394		0.984	0.02	0.997	0.09	0.99	8.9E-01
50855	PARD6A	A_23_P140821		0.768	0.03	0.785	0.15	0.98	9.2E-01
2889	RAPGEF1	A_23_P391764	Probe 1	1.281	0.02	1.313	0.05	0.98	5.5E-01
25	ABL1	A_24_P291016	Probe 4	0.986	0.01	1.020	0.04	0.97	4.3E-01
5359	PLSCR1	A_23_P69109		3.743	0.12	3.892	0.57	0.96	8.1E-01
7071	KLF10	A_23_P168828		1.025	0.03	1.125	0.21	0.91	6.5E-01
4739	NEDD9	A_23_P344555		1.035	0.05	1.194	0.20	0.87	4.7E-01
1950	EGF	A_23_P155979		0.531	0.03	0.619	0.05	0.86	1.4E-01
25	ABL1	A_24_P393711	Probe 3	0.734	0.01	0.867	0.04	0.85	1.9E-02
309	ANXA6	A_23_P357104		0.368	0.01	0.460	0.04	0.80	6.4E-02
8655	DYNLL1	A_24_P124672	Probe 2	1.502	0.02	1.979	0.22	0.76	7.2E-02
5578	PRKCA	A_24_P916496	Probe 2	0.447	0.02	0.606	0.07	0.74	4.7E-02
25	ABL1	A_23_P60180	Probe 2	0.720	0.01	0.979	0.04	0.74	9.4E-05
25	ABL1	A_24_P281101	Probe 1	0.666	0.01	0.909	0.04	0.73	2.3E-04
2036	EPB41L1	A_23_P154806	Probe 2	1.606	0.04	2.193	0.20	0.73	2.1E-02
2889	RAPGEF1	A_23_P304237	Probe 2	0.453	0.01	0.699	0.05	0.65	1.1E-03
8655	DYNLL1	A_23_P65031	Probe 1	1.440	0.02	2.266	0.23	0.64	7.9E-03
5159	PDGFRB	A_23_P421401		3.072	0.12	5.205	1.10	0.59	9.5E-02
2037	EPB41L2	A_23_P134109		1.207	0.05	2.200	0.38	0.55	3.4E-02
5063	PAK3	A_23_P346813		1.433	0.09	3.623	0.71	0.40	1.7E-02
